# Enzyme catalyzes ester bond synthesis and hydrolysis: The key step for sustainable usage of plastics

**DOI:** 10.3389/fmicb.2022.1113705

**Published:** 2023-01-12

**Authors:** Jinghui Lai, Huiqin Huang, Mengwei Lin, Youqiang Xu, Xiuting Li, Baoguo Sun

**Affiliations:** ^1^Key Laboratory of Brewing Microbiology and Enzymatic Molecular Engineering of China General Chamber of Commence, Beijing Technology and Business University, Beijing, China; ^2^Key Laboratory of Brewing Molecular Engineering of China Light Industry, Beijing Technology and Business University, Beijing, China

**Keywords:** plastics, bio-plastics, plasticizers, enzyme, ester bond, recycling

## Abstract

Petro-plastic wastes cause serious environmental contamination that require effective solutions. Developing alternatives to petro-plastics and exploring feasible degrading methods are two solving routes. Bio-plastics like polyhydroxyalkanoates (PHAs), polylactic acid (PLA), polycaprolactone (PCL), poly (butylene succinate) (PBS), poly (ethylene furanoate) s (PEFs) and poly (ethylene succinate) (PES) have emerged as promising alternatives. Meanwhile, biodegradation plays important roles in recycling plastics (e.g., bio-plastics PHAs, PLA, PCL, PBS, PEFs and PES) and petro-plastics poly (ethylene terephthalate) (PET) and plasticizers in plastics (e.g., phthalate esters, PAEs). All these bio- and petro-materials show structure similarity by connecting monomers through ester bond. Thus, this review focused on bio-plastics and summarized the sequences and structures of the microbial enzymes catalyzing ester-bond synthesis. Most of these synthetic enzymes belonged to α/β-hydrolases with conserved serine catalytic active site and catalyzed the polymerization of monomers by forming ester bond. For enzymatic plastic degradation, enzymes about PHAs, PBS, PCL, PEFs, PES and PET were discussed, and most of the enzymes also belonged to the α/β hydrolases with a catalytic active residue serine, and nucleophilically attacked the ester bond of substrate to generate the cleavage of plastic backbone. Enzymes hydrolysis of the representative plasticizer PAEs were divided into three types (I, II, and III). Type I enzymes hydrolyzed only one ester-bond of PAEs, type II enzymes catalyzed the ester-bond of mono-ester phthalates, and type III enzymes hydrolyzed di-ester bonds of PAEs. Divergences of catalytic mechanisms among these enzymes were still unclear. This review provided references for producing bio-plastics, and degrading or recycling of bio- and petro-plastics from an enzymatic point of view.

## Introduction

Plastics were ubiquitous and indispensable in the modern society since 1950s (Geyer et al., 2017). Due to their flexibility, strength, versatility, durability and low cost, the global plastics production increased exponentially, from 2 million tons in 1950s to more than 380 million tons per year now, with production expected to double in the next 20 years (Viljakainen and Hug, 2021; Gambarini et al., 2022). The extensive use of plastics unavoidably generated a remarkable amount of plastic waste and caused a serious plastic pollution that required to be settled urgently (Geyer et al., 2017). It was reported that approximately 6,300 million metric ton of plastic waste had been generated until 2015 (Geyer et al., 2017). There were two sources of raw materials for producing these plastics, petro-based and bio-based materials (Tang et al., 2012). The plastics market was dominated by those low-cost petro-plastics, including polyethylene terephthalate (PET), polyethylene (PE), polyvinyl chloride (PVC), polypropylene (PP), and polystyrene (PS; Chamas et al., 2020). However, these materials were resistant to degradation and persisted for a long time in the environment, and caused serious plastic pollution (Webb et al., 2012; Duis and Coors, 2016; Chamas et al., 2020). Furthermore, “microplastics,” the tiny plastic pieces smaller than 5 mm in size, also posed a great threat to environment and human health (Qin et al., 2021). Studies showed that microplastics could change the distribution pattern and bio-accessibilities of persistent organic pollutants in soil due to microplastic’s persistency and large specific surface area (Qin et al., 2021), and affected the composition of freshwater benthic communities and brought negative effects to the whole biosphere (Redondo-Hasselerharm et al., 2020).

Additionally, plasticizers were another kind of chemicals urgently needed to be treated (Xu et al., 2020). Plasticizers were often added to petro-plastics to improve the properties of products, such as flexibility and durability to meet industrial requirements (Katsikantami et al., 2016). Phthalate esters (PAEs) were the representative of plasticizers, which were one kind of di-ester compounds formed by esterification of alcohols with the two carboxyl groups of phthalic acid (Katsikantami et al., 2016; Xu et al., 2020). PAEs accounted for more than 55% of global plasticizer market in 2020.^1^ In plastics, the amount of PAEs added was relatively in a large amount, for example, the amount of PAEs added in PVC plastics could reach more than 40% (Graham, 1973). Recent studies found that PAEs acted as endocrine disruptors, and brought serious negative effects to human health by causing abnormal lipid metabolism, childhood obesity, immune response interference, and neuropsychological disease (Xu et al., 2020). According to the latest assessment by the European Food Safety Agency in 2019, the limitation of human tolerance to PAEs was lower than 50 μg/(Kg·d) (Xu et al., 2021b). As there were no covalent bonds between PAEs and the plastic backbone, PAEs were easily escaped into the environment, such as farmland and water (Xu et al., 2021b). Studies found that the concentrations of PAEs in some surface water and soil were as high as 500 mg/l and 63 mg/Kg, respectively (Net et al., 2015). Meanwhile, crops could accumulate PAEs in the seeds, such as rice and wheat, and caused food contamination (Ma et al., 2013; Sun et al., 2015). Therefore, during plastic treatment, plasticizers in plastics was another kind of serious pollutant needed to be carefully considered.

Plastic recycling was one way to address current plastic pollution. One commonly used recycling method was mechanical recycling, which transformed plastics into raw materials through some mechanical handling, such as washing, shredding, melting and reshaping. However, the quality of the plastic materials obtained by this method went from bad to worse (Ragaert et al., 2017). Another way was chemical recycling, which could produce higher quality products but with significantly higher economical cost and larger energy input (Ragaert et al., 2017). Bio-recycling was a promising method depending on the enzymatic degradation of plastics. The degraded monomers could be used as building blocks to manufacture new plastics or other higher value compounds in a circular economic manner (Zhu et al., 2022). The chemical bonds found in plastics generally were ester bonds, urethane bonds, ether bonds and carbon–carbon bonds, thereinto, ester bonds were common and easily be hydrolyzed (Stubbins et al., 2021; Verschoor et al., 2022). One of the most commonly used and representative plastics, PET, attracted great attention by the enzymatic recycling studies through hydrolyzing the ester bonds of the backbone to realize biodegradation (Lu et al., 2022). However, for other kinds of petro-plastics with non-ester bonds linked monomers, biodegradation was still a bottleneck to be solved.

Bio-plastics, a promising alternative to petro-plastics, could be finally degraded to carbon dioxide, water, and biomass through microbial mineralization (Othman, 2014; Lambert and Wagner, 2017). Studies showed that the carbon from each monomeric unit of bio-plastic poly(butylene adipate-co-terephthalate) (PBAT) was used by microorganisms, such as filamentous fungi, to obtain energy and form biomass (Thomas et al., 2018). Meanwhile, bio-plastics could serve some new applications related to human health, for example, they were used in medical industries because of their good processability, biocompatibility, and non-toxicity (Grigore et al., 2019). All these bio-plastics showed a similar structural characteristic that the monomers were mostly linked by ester bonds to produce the polymers (Adrah et al., 2020).

Based on the structures of above petro-/bio- plastics or the plasticizer PAEs, a common point in structure was the ester bond, which was crucial for biodegradation of PET or PAEs and biosynthesis or degradation of bio-plastics. Therefore, this review summarized the bio-plastics synthesizing and degrading enzymes, including the sequences, structures and catalytic properties of these enzymes. The sequences, structures and catalytic properties of PET ester-bond hydrolases and PAEs ester-bond hydrolases were also investigated. Meanwhile, the current challenges and future perspectives of plastic recycling were discussed from the enzymatic perspective.

## Materials for producing bio-plastics

Developing bio-plastics, an alternative to petro-plastics, was one promising way to deal with petro-plastic pollution. The materials for producing bio-plastics included bio-macromolecules and small molecule monomers (Adrah et al., 2020). Bio-macromolecules like starch, cellulose and protein were the materials for thermoplastic starch, cellulose-based plastics, and protein-based plastics, respectively, and they could be obtained from renewable resources (mainly plants; Adrah et al., 2020). Recent studies also tried to develop plastic polymers using DNA as the raw materials (Han et al., 2021). However, these materials were generally not appropriated to use directly in the natural form, and needed to be modified by blending with other copolymers to show the properties of plastics, thus limited their application (Adrah et al., 2020). Therefore, these materials were not discussed in this review.

The small molecule monomers and their corresponding bio-polymers were shown in Table 1. These small monomers mainly included the molecules with one hydroxyl group and one carboxyl group, diols, dicarboxylic acids and cyclic monomers. Most of monomers could be obtained by biocatalytic methods. For example, ε-caprolactone, the monomer of polycaprolactone (PCL), could be obtained by the oxidation of cyclohexanone with the cyclohexanone monooxygenase from *Acinetobacter calcoaceticus* as the catalyst (Pyo et al., 2020). Glycolic acid, the monomer of poly(glycolic acid) (PGA), was produced from glycolonitrile using the nitrilase as catalyst in aqueous condition (Jem and Tan, 2020). As monomers of poly(ethylene furanoate) (PEF), ethylene glycol and 2,5-furandicarboxylic acid (2,5-FDCA) could also be obtained through biocatalysis. Ethylene glycol could be synthesized using glycerol as the raw material through the artificial enzymatic cascade containing alditol oxidase, catalase, glyoxylate/hydroxypyruvate reductase, pyruvate decarboxylase and lactaldehyde:propanediol oxidoreductase (Li et al., 2021). FDCA could be obtained using 5-hydroxymethylfurfural (HMF) as the raw material catalyzed by 5-hydroxymethylfurfural oxidase and Novozym 435 (Wu et al., 2020). Although some monomers were obtained by chemical reaction, they might also be produced by biocatalytic methods in future. For example, 1,3-propanediol was previously produced using 1,2-ethanediol and acrolein from petroleum oil through chemical process, but now it could be produced through microbial cells such as engineered *E. coli*, *Clostridium butyricum*, and *Klebsiella pneumoniae* (Lee et al., 2018; Lama et al., 2020; Lan et al., 2021).

**Table 1:** Structure and production methods of bio-plastics and their materials.

Bio-plastics	Materials	Materials production	The production method of polymer	Reference
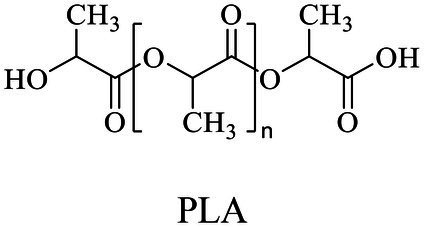	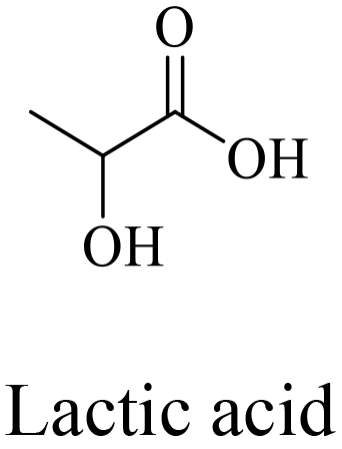	Biocatalytic	Polycondensation or ring-opening polymerization (ROP) (metal-based or enzyme catalysis)	Havstad (2020), Narayanan et al. (2004)
		Chemical		
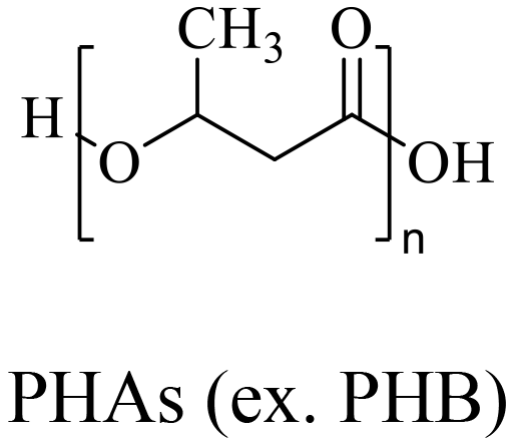	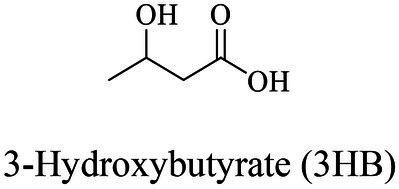	Biocatalytic	Metabolic synthesis	Dalton et al. (2022), Agostini et al. (1971), Vroman and Tighzert (2009)
	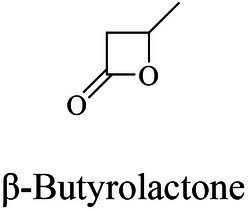	Chemical	ROP (metal-based or enzyme catalysis)	
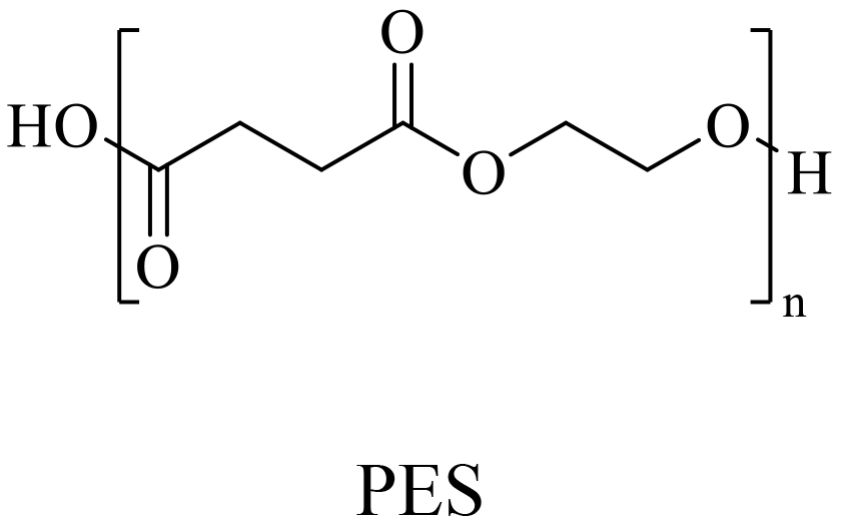	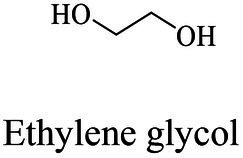	Chemical/Biocatalytic	Polycondensation or ROP (enzyme catalysis)	Maihom et al. (2008), Li et al. (2021), Xu and Guo (2010), Morales-Huerta et al. (2017), Chen et al. (2009)
	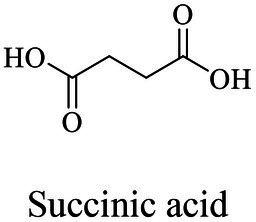	Chemical/Biocatalytic		
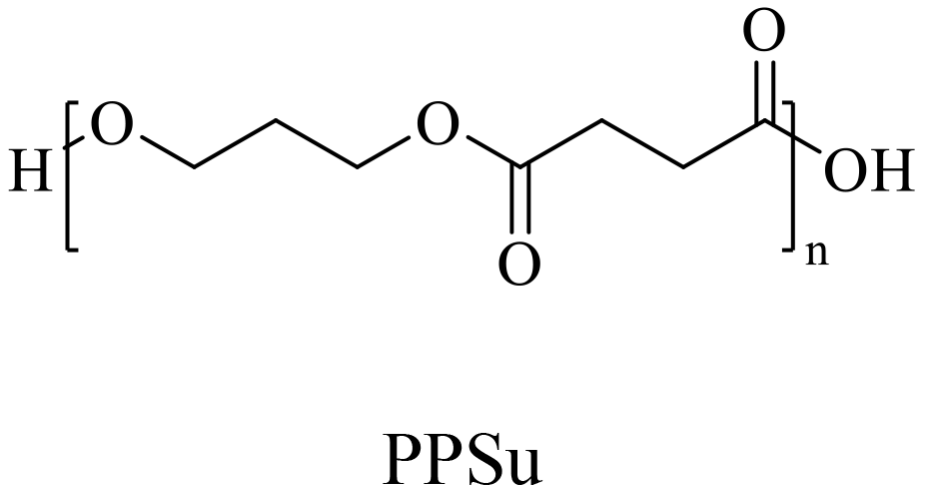	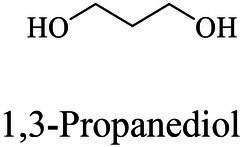	Chemical/ Biocatalytic	Polycondensation	Kraus (2008), Lee et al. (2018), Bikiaris et al. (2009)
	Succinic acid	Same as PES		
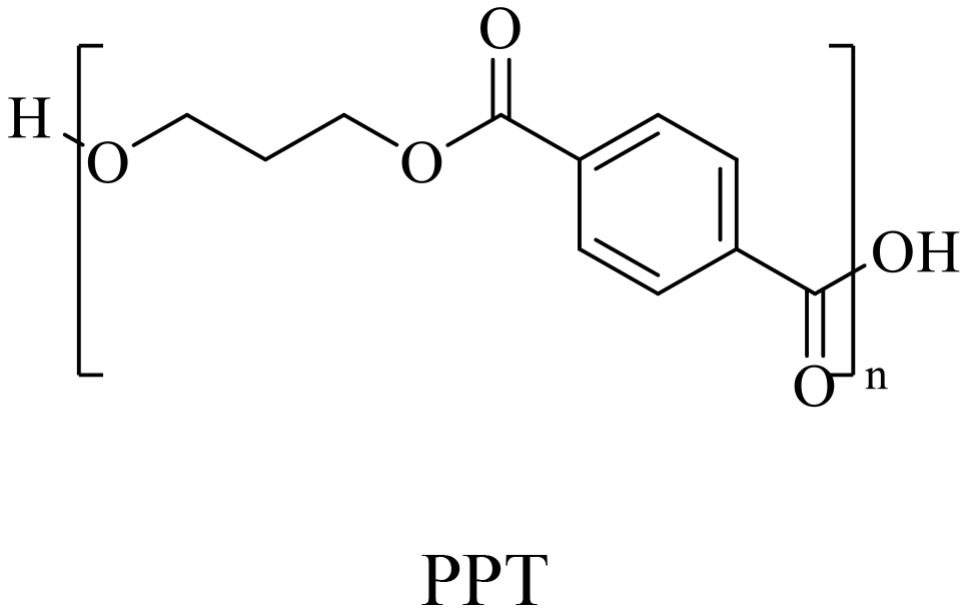	1,3-Propanediol	Same as PPSu	Polycondensation	Morgan et al. (2002), Collias et al. (2014), Karayannidis et al. (2003)
	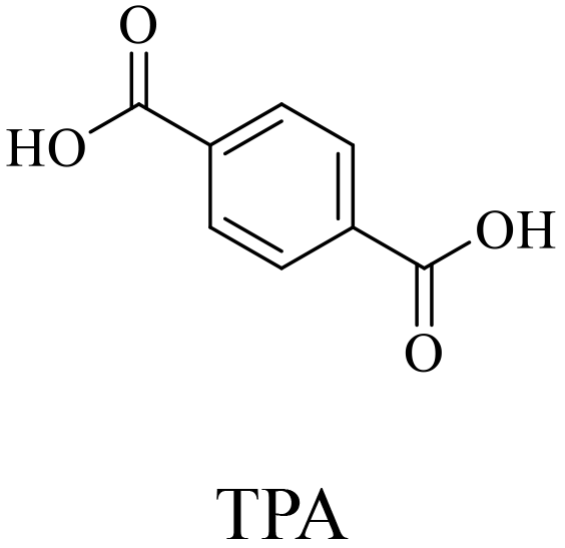	Chemical/ Biocatalytic		
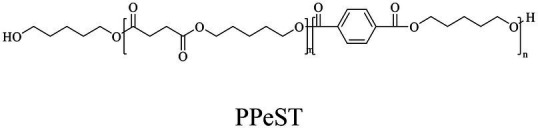	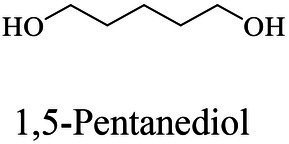	Chemical/Biocatalytic	Copolycondensation	Lu et al. (2019), Wijaya et al. (2017), Cen et al. (2020)
	Succinic acid	Same as PES		
	Terephthalic acid	Same as PPT		
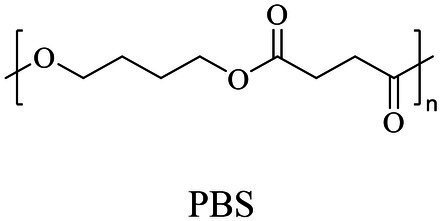	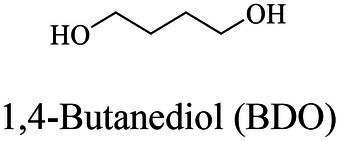	Biocatalytic	Polycondensation/ROP (metal-based or enzyme catalysis)	Peñas et al. (2022), Xu and Guo (2010)
		Chemical		
	Succinic acid	Same as PES		
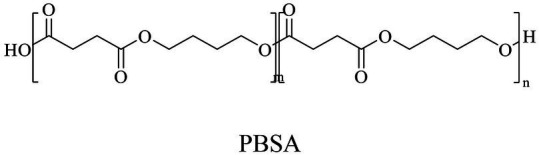	BDO	Same as PBS	Copolycondensation	Pivsa-Art et al. (2011), Lang and Li (2022)
	Succinic acid	Same as PES		
	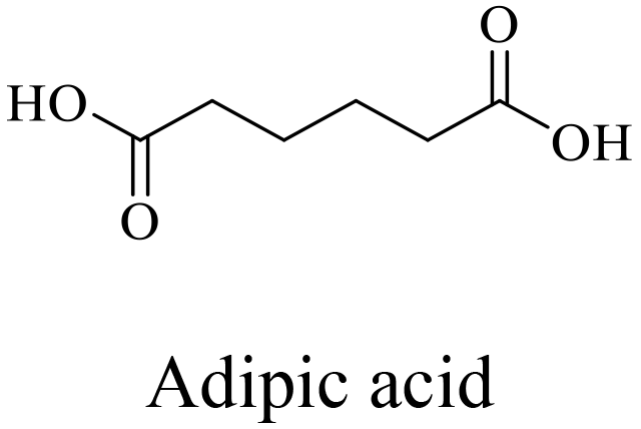	Chemical/Biocatalytic		
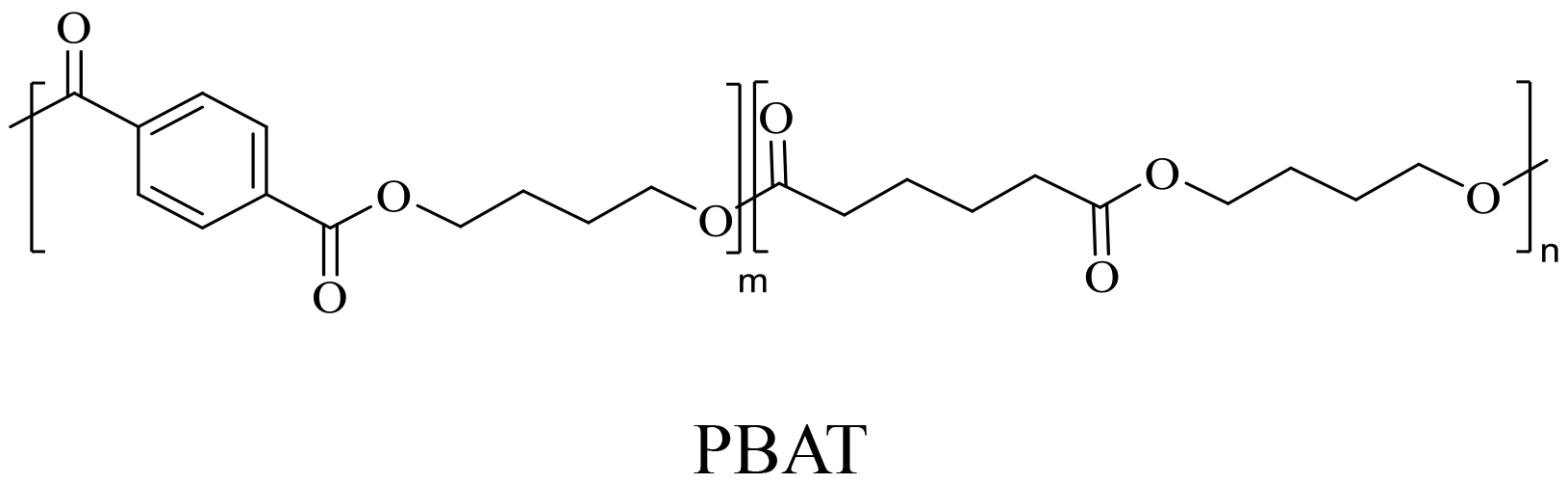	Adipic acid	Same as PBSA	Polycondensation	Deng et al. (2016), Vroman and Tighzert (2009), Lang and Li (2022), Pang et al. (2016), Collias et al. (2014)
	Terephthalic acid	Same as PPT		
	BDO	Same as PBS		
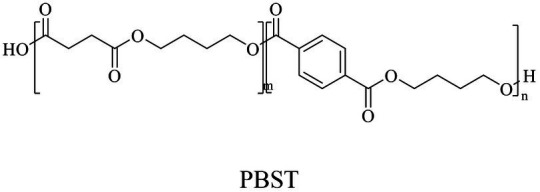	Terephthalic acid	Same as PPT	Polycondensation	Zheng et al. (2021)
	BDO	Same as PBS		
	Succinic acid	Same as PES		
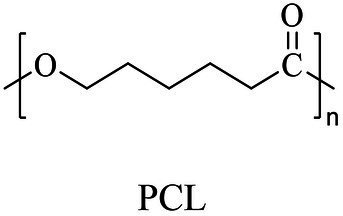	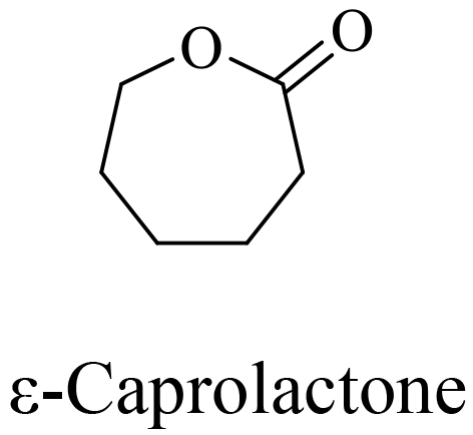	Chemical	ROP (metal-based or enzyme catalysis)	Goyal et al. (2022), Pyo et al. (2020), Vroman and Tighzert (2009), Figueiredo et al. (2020)
		Biocatalytic		
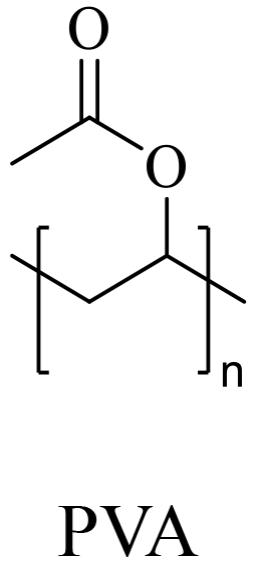	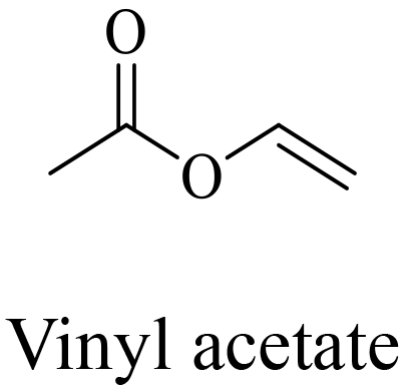	Chemical	Polymerization and hydrolysis	Caranton et al. (2020), Labet and Thielemans (2009)
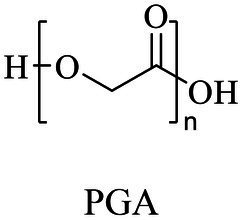	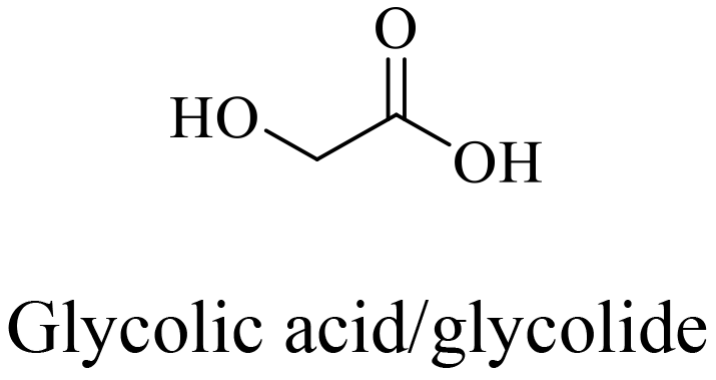	Chemical	Polycondensation or ROP	Jem and Tan (2020)
		Biocatalytic		
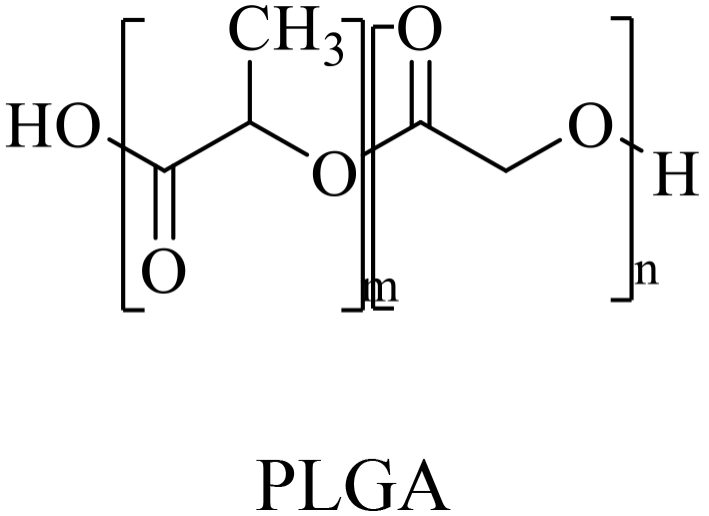	Glycolic acid/glycolide	Same as PGA	ROP (metal-based or enzyme catalysis)	Chanfreau et al. (2010), Dechy-Cabaret et al. (2004)
	Lactic acid	Same as PLA		
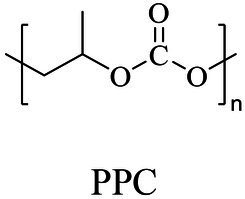	Carbon dioxide	Chemical	Copolymerization	Li et al. (2022), Zhang et al. (2017), Lee et al. (2018)
		Biocatalytic		
	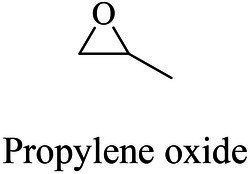	Chemical		
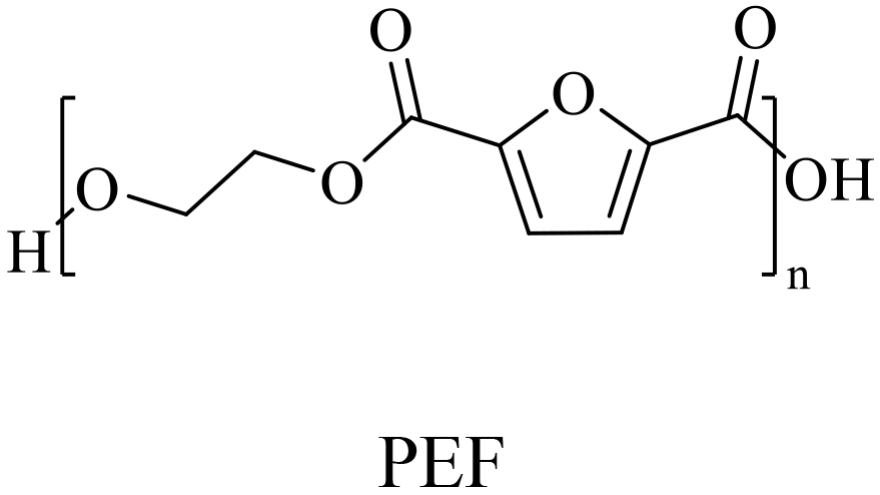	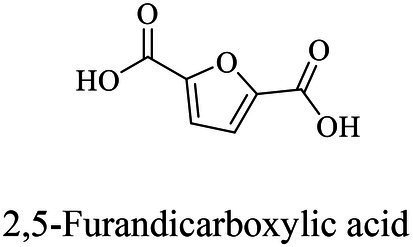	Chemical/ Biocatalytic	Polycondensation/ROP (non-metal/metal-based or enzyme catalysis)	Gorbanev et al. (2009), Wu et al. (2020), Maihom et al. (2008), Li et al. (2021), Loos et al. (2020), Tong et al. (2010)
	Ethylene glycol	Same as PES		
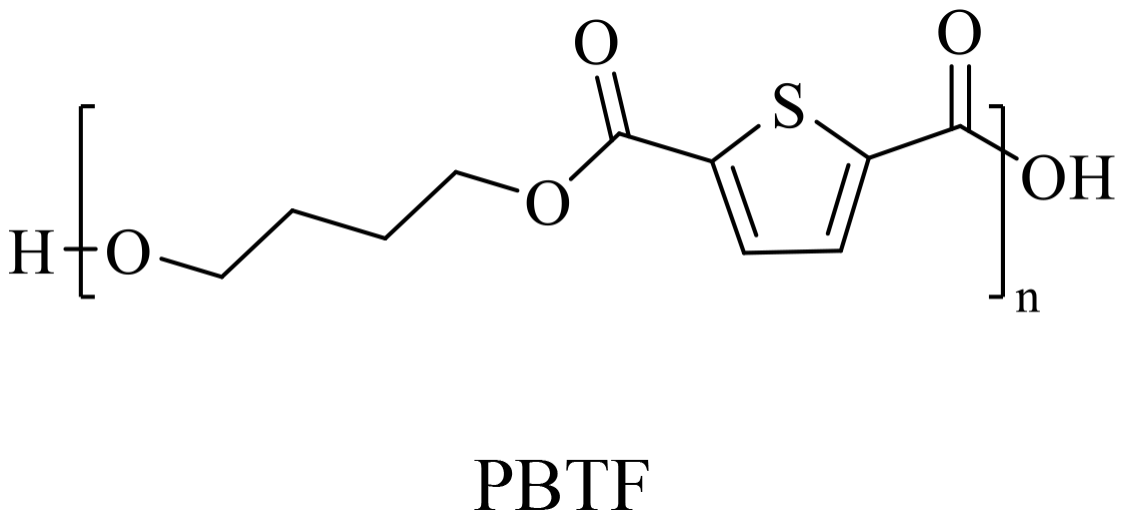	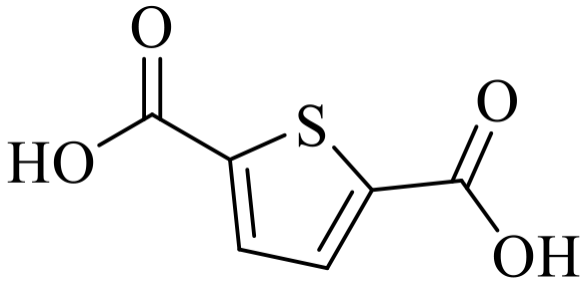 2,5-Thiophenedicarboxylic acid (TDCA)	Chemical	Polycondensation	Wang et al. (2022), Wang et al. (2019)
	BDO	Same as PBS		

In general, the monomers could be polymerized into bio-polymers *via* biological catalysis and chemical polymerization. The chemical esterification method produced polymers including polypropylene carbonate (PPC), polyvinyl alcohol (PVA), Poly(butylene succinate-co-butylene adipate) (PBSA), PBAT, poly(butylene succinate-co-butylene terephthalate) (PBST) and poly(butylene 2,5-thiophenedicarboxylate) (PBTF). Among them, it was worth noting that PBTF, a possible alternative to FDCA-polyester, was received great attention in recent years due to its exceptionally high gas barrier properties (Guidotti et al., 2018). Studies reported that thiophenedicarboxylic acid (TDCA)-polyester showed similar or even better gas barrier performance as FDCA-polymers, but related industrial applications needed to be further developed (Guidotti et al., 2018). Biological catalysis converted monomers into polymers through formation of ester bonds with enzymes as catalysts. For example, polyhydroxyalkanoates (PHAs), polylactic acid (PLA), poly (butylene succinate) (PBS), PCL, and PES, the commercialized plastics (Ghorpade et al., 2022), could be synthesized by enzyme catalysis (Table 1). Recently, studies also developed novel materials and among them, PEF was one of the representatives. It performed higher barrier and heat resistance than that of PET (Debuissy et al., 2018), and could replace PET in the fields of food packaging, personal and home care containers and thermoforming equipments (Pellis et al., 2016). PEF could be synthesized from 2,5-FDCA and ethylene glycol by metal or organic non-metallic catalysts (Morales-Huerta et al., 2016), and also be catalyzed by the *Candida antarctica* lipse B (CALB; Nasr et al., 2022). Since this review mainly focused on the summary of enzyme catalyzed ester bond synthesis and hydrolysis of related polymers, the following part focused on the representative bio-polymers PHAs, PLA, PBS, PCL, PEF and PES, and fully discussed the sequence and structural characteristics of related enzymes. The summary could provide the enzymology reference and engineering strategy for the efficient production of bio-polymers.

## Enzyme catalyzes the ester bond synthesis of bio-plastics

Enzymes catalyzing the synthesis of bio-plastics were summarized in Table 2, mainly involving PHA synthases (PhaCs) and lipases. In general, the sequence of an enzyme determines its structure, which in turn affects the catalytic property. Therefore, this part discussed the amino acid sequence and structure of these enzymes.

**Table 2:** Synthesis of bio-plastics by enzymes from different microbial sources.

Bio-plastics	Substrate	Enzyme type	Source	Synthesis method	Reference
PHAs					
PHA	Specific for short and medium chain length 3HA units	PHA synthases 1	*Pseudomonas* sp. 61–3	Expression in recombinant	Matsusaki et al. (1998)
P(3HB)	Specific for short chain length	PHB synthase (PhbC_Ps_)	*Pseudomonas* sp. 61–3	Expression in recombinant	Matsusaki et al. (1998)
PHA	–	PhaC1	*Pseudomonas mendocina*	Expression in recombinant	Hein et al. (2002)
P(3HB)	–	PHA synthases	*Delftia acidovorans* DS-17	Expression in recombinant	Hiroe et al. (2013)
PHA	–	PHA synthases (PhaC1_HO1_)	*Halomonas* sp. O-1	Expression in recombinant	Ilham et al. (2014)
PHA	–	PHA synthase (PhaC1_He_)	*Halomonas elongata* DSM2581		Ilham et al. (2014)
PHA	Only mcl 3HA	PHA synthase (PhaC1_Ps_)	*Pseudomonas stutzer*i strain 1,317	Expression in recombinant	Chen et al. (2006)
PHA	Scl/mcl 3-hydroxybutyrate	PHA synthase (PhaC2_Ps_)			Chen et al. (2006)
P3HB	[^14^C]-HB-CoA	D302A-PhaCPhaE	*Allochromatium Vinosum*	*In vitro*, 30°C	Tian et al. (2005)
					Jia et al. (2000)
PHA	HB-CoA	PHA synthase (PhaC_Bm_)	*Bacillus megaterium*	*in vivo* or *in vitro*	McCool and Cannon (2001)
P(3HB)	(R,S)-β-butyrolactone (BL)	(PHB) depolymerase	*Pseudomonas stutzeriwas*	*in vitro*(ROP)	Suzuki et al. (2000)
P(3HB)	β-Butyrolactone	Lipase (ESL-001, Lipase M)	commercial	*in vitro*(ROP)	Xie et al. (1997)
					Matsumura et al. (1998)
PLA					
PLA (2600–4,500 Da)	Lactic acid	Lipase (Lipozyme TL IM)	*Thermomyces lanuginosus*	*In vitro*, toluene, 50°C, 5 h	Chuensangjun et al. (2012)
PLA (26,000 Da)	L-Lactide	Lipase B (Novozym 435)	*Candida antarctica*	Toluene, at 80°C	Omay and Guvenilir (2013)
					Uppenberg et al. (1994)
PLA	Lactide	Lipase M	*Mucor javanicus*	*In vitro*, toluene, 40°C for 24 h	Yadav et al. (2021)
LA-based copolyesters	ε-Caprolactone and D,L-lactide	Lipase B	*Candida antarctica*	*In vitro* (ROP)	Kumar et al. (2000), Matsumoto and Taguchi (2010)
PLA	L, L-/D,D-lactides	Lipase B (Novozym 435)	–	*In vitro*	Matsumoto and Taguchi (2010)
PLA (37,800 Da)	L-LA	Lipase B (Novozym 435)	*Candida antarctica*	*In vitro*; 65°C	Chanfreau et al. (2010)
P(LA-co-3HB)	–	LA-polymerizing enzyme	*Pseudomonas* sp. 61–3	whole-cell synthesis	Song et al. (2012)
PLA	L-lactide	Lipase	*Candida rugosa*	*In vitro*, 90°C, 2% (w/w) lipase	Rahmayetty et al. (2018)
					Domínguez de María et al. (2006)
PLGA(Poly (lactic-co-glycolic acid) (PLGA))	L-Lactide and glycolide	Lipase B (Novozym 435)	*Candida antarctica*	*In vitro*, 65°C in [HMIM][PF_6_]	Chanfreau et al. (2010)
PCL					
PCL	ε-Caprolactone	Lipase B	*Candida antarctica*	*In vitro*, less than 10 h in bulk at 60°C	Kobayashi (2009)
PCL	ε-Caprolactone	Cutinase (HiC)	*Humicola insolens*	*In vitro*, 0.1% (w/w) HiC at 70°C in bulk for 24 h	Hunsen et al. (2007), Kold et al. (2014)
PCL (7,000 Da)	ε-Caprolactone	Lipase	*Pseudomonas fluorescens*	*In vitro*, 60°C in bulk for 10 days	Uyama and Kobayashi (1993)
PCL	ɛ-Caprolactone	Lipase	*Fervidobacterium nodosum* Rt17-B1	*In vitro*, 90°C in toluene for 72 h	Li et al. (2011), Yu et al. (2010)
PCL	ɛ-Caprolactone	Carboxylesterase	*Archaeoglobus fulgidus*	*In vitro*, 80°C in toluene for 72 h	Ma et al. (2009)
PBS					
PBS (130,000 Da)	Dimethyl succinate and butane-1,4-diol	Lipase CA	*Candida antarctica*	*In vitro*, in toluene	Sugihara et al. (2006)
PBS (73,000 Da)	Succinic anhydride, butane-1,4-diol, and succinic acid	Lipase B (Novozym 435)	*Candida antarctica*	*In vitro*	Ren et al. (2015)
PEFs	Dimethyl 2,5-furandicarboxylate (DMFDCA) and aliphatic diols	Lipase B (Novozym 435)	*Candida antarctica*	*In vitro*, 80°C in diphenyl ether	Jiang et al. (2015)
PES (60,000 Da)	Cyclic oligo(ethylene succinate)s	Lipase B (Novozym 435)	*Candida antarctica*	*In vitro*, 125°C in toluene	Morales-Huerta et al. (2017)

“–” means not found in literature.

### Sequences, structures and properties of enzymes for synthesis of PHAs

PHAs were aliphatic polyesters produced by various microorganisms under unbalanced growth condition (Stubbe and Tian, 2003; Tsuge et al., 2015). PHAs were mainly synthesized by PhaCs using 3-hydroxyalkanoates-coenzyme A (3HAs-CoA) as the substrate (Sagong et al., 2018). Studies also reported β-butyrolactone could be polymerized into polyhydroxybutyrate (PHB) using the PHB depolymerase from *Pseudomonas stutzeriwas* or commercial lipases as catalysts (Matsumura et al., 1998; Suzuki et al., 2000; Table 2). However, the studies on these enzymes were relative rare, and hardly any studies reported about the structures of these enzymes. Therefore, the following paragraphs focused on PhaCs.

According to the reported pathways of PHA synthesis in microorganisms, acetyl-CoA was an important precursor. Among various PHAs, the synthetic pathway of PHB was firstly confirmed. Two acetyl-CoAs were condensed into acetoacetyl-CoA catalyzed by acetyl-CoA acetyltransferase (β-ketothiolase, PhaA), and then acetoacetyl-CoA was converted into (*R*)-3-hydroxybutyryl-CoA [(*R*)-3-HB-CoA] by acetoacetyl-CoA reductase (PhaB). Finally, (*R*)-3-HB-CoA was polymerized into PHB by the PhaC (Sagong et al., 2018). As referred above, PhaC played a crucial role for producing polymers. Therefore, the sequences and structures of PhaC were analyzed to provide a theoretical basis for enzymatic engineering to improve the catalytic efficiency of PhaC in future works.

As shown in Table 2, PhaCs were able to catalyze the polymerization of HAs-CoA to PHAs *in vitro*, which started polymerization with HAs-CoA as the substrate (Sagong et al., 2018). For example, PhaC from *Bacillus megaterium* could catalyze the polymerization of HB-CoA to PHA *in vitro* (McCool and Cannon, 2001). Using the D302A mutant of PHB synthase from *Allochromatium Vinosum* as the catalyst, P3HB was synthesized with HB-CoA as the substrate *in vitro*. As shown in Table 2, PhaCs showed different substrate specificities when catalyzed the synthesis of PHAs. High molecular weight poly [(*R*)-3-HB] was synthesized by PHA syntheses (PhaC_Da_) from *Delftia acidovorans* DS-17 (Hiroe et al., 2013). Similarly, the synthesis of a random copolymer poly(3-hydroxybutyrate-co-3-hydroxyalkanoate) was realized by PhaC from *Pseudomonas* sp. strain 61–3, which showed substrate preference toward scl-3HA (with 3–5 carbons in the monomer) and mcl-3HA units (with 6–14 carbons in the monomer; Matsusaki et al., 1998). PhaC1_Ps_ and PhaC2_Ps_, derived from *P. stutzeri* strain 1317, were both with the ability to synthesize PHAs (Chen et al., 2006), whereas, PhaC1_Ps_ only catalyzed the synthesis of mcl-3HA, and PhaC2_Ps_ could catalyze the polymerization of both scl-3HA and mcl-3HA (Chen et al., 2006).

**Figure 1:**
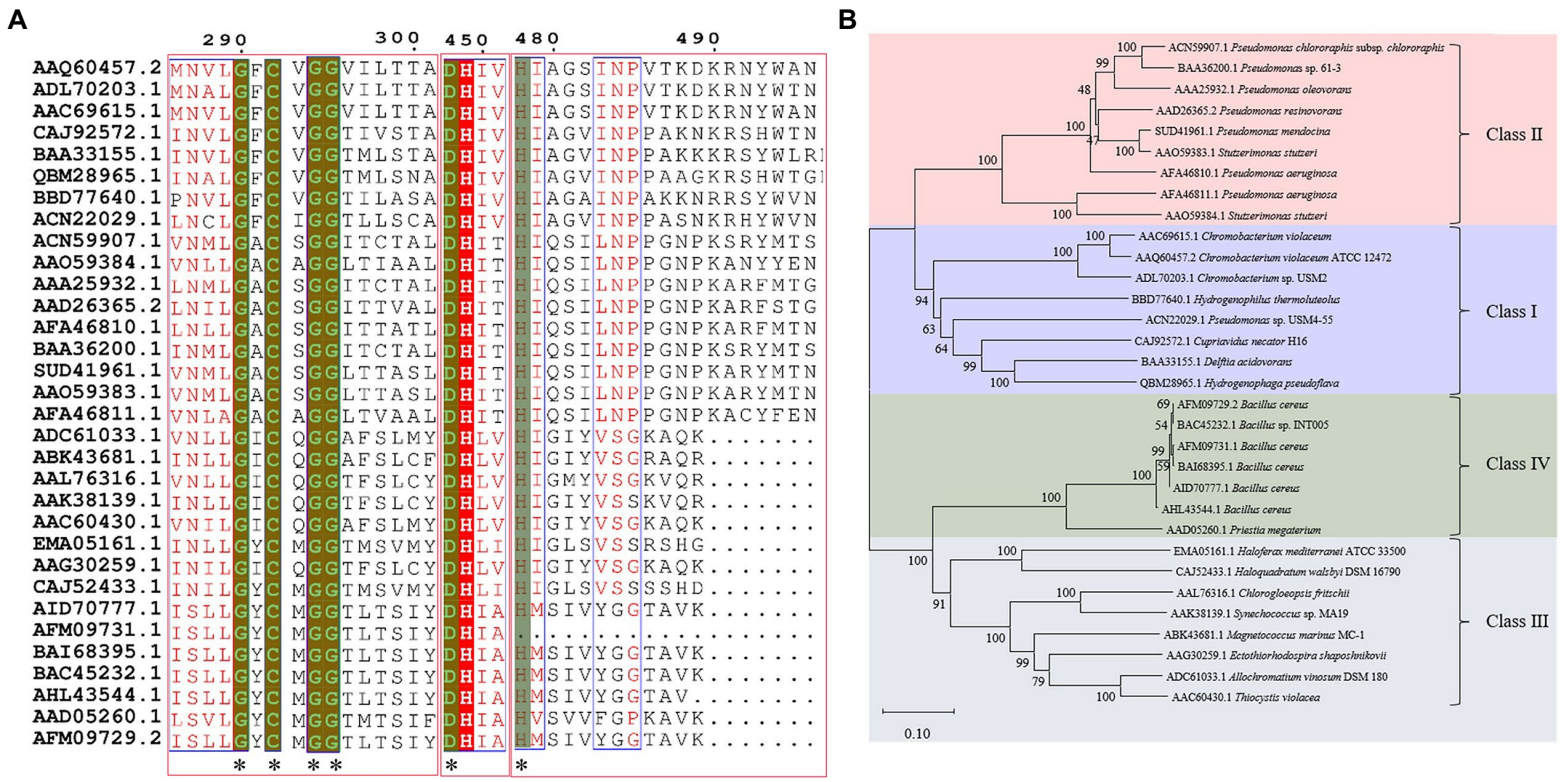
Sequence alignment and phylogenetic tree of PHA synthases. **(A)** Sequence alignment of PHA synthases. “*” Conservative amino acid. **(B)** Phylogenetic tree of PHA synthase.

In previous studies, PHA synthases were divided into four classes (Class I, Class II, Class III and Class IV) according to their sequences and substrate specificities (Rehm, 2003). It was generally recognized that Class I, Class III and Class IV PhaCs preferred to use scl-3HA-CoAs as the substrate, while Class II generated PHAs with mcl-3HA-CoAs as the substrate (Neoh et al., 2022). However, the Class IV synthase resources were relatively rare, only two sequences were analyzed in previous works (Rehm, 2003). In this review, Class IV PHA synthases were supplemented, and sequence alignment analysis was performed of 32 PHA synthases that were verified to show PHAs synthesis abilities. Results were shown in Figure 1A, with sequence identity between 23 and 100%, and carried conservative sequence G-X-C-X-G (where X was an arbitrary amino acid). Based on multiple sequence alignment, the phylogenetic tree was constructed for these 32 PhaCs (Figure 1B). All the PhaCs were divided into four classes, which was consistent with the current classification of PhaCs (Rehm, 2003).

**Figure 2:**
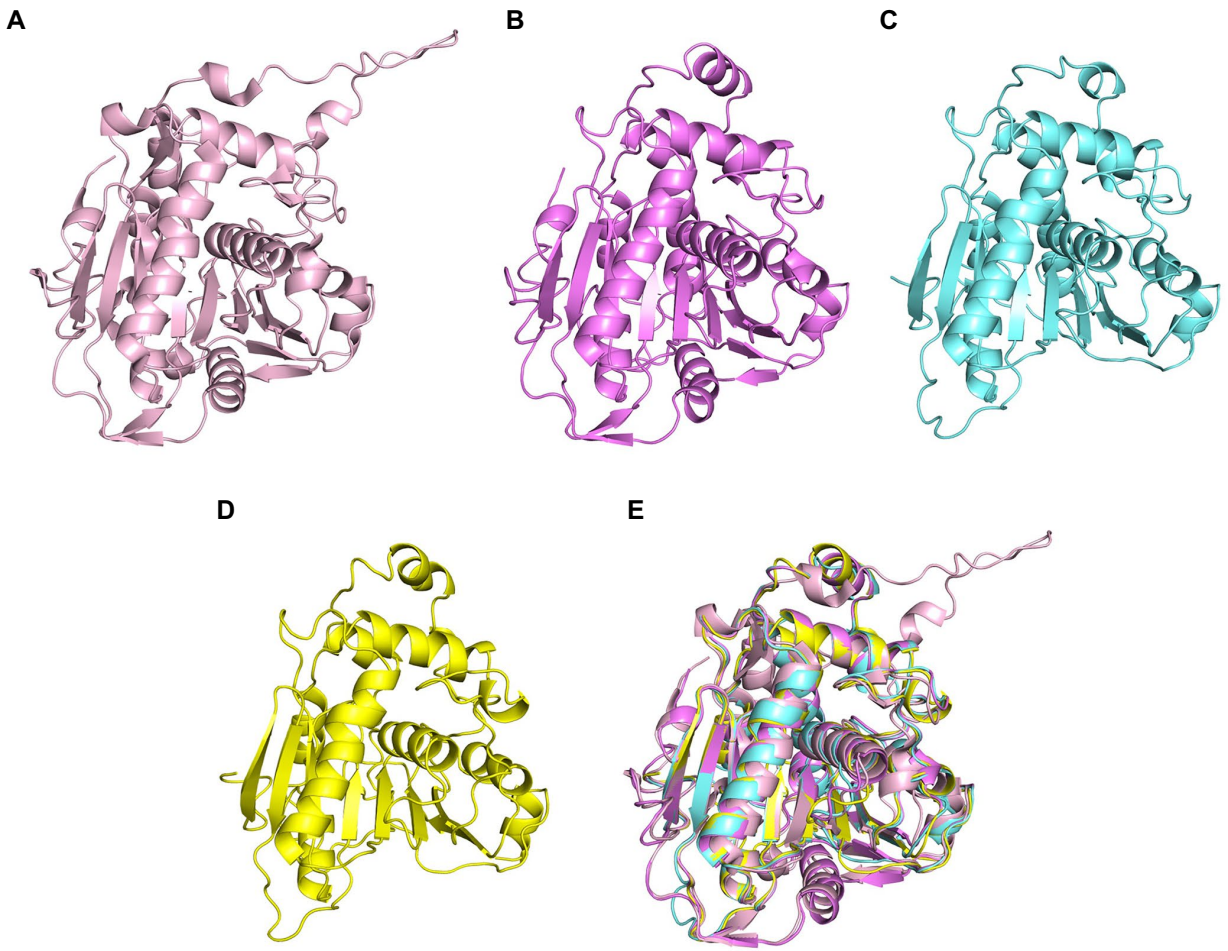
Homologous modeling of PHA synthases. **(A)** Class I PHA synthase (*Cupriavidus necator* H16), **(B)** Class II PHA synthase (*Pseudomonas* sp. 61-3) **(C)** Class III PHA synthase (*Allochromatium vinosum* DSM 180) **(D)** Class IV PHA synthase (*Bacillus* sp. INT005) **(E)** structure alignment of these PHA synthase.

Compared with many reported sequences of PhaCs, studies about the structures of PhaC were rare. Only two studies reported crystal structures of Class I PhaCs to date, and the two enzymes were derived from *Cupriavidus necator* H16 (Protein Data Bank (PDB) ID 5HZ2) and *Chromobacterium* sp. USM2 (PDB ID 5XAV), respectively. Sequences of the two enzymes showed the identity of 51.79%. Based on this, the representative PhaCs from Class II, Class III, and Class IV were selected for homologous modeling with GenBank ID BAA36200.1 (*Pseudomonas* sp. 61-3), ADC61033.1 (*A. vinosum* DSM 180), and BAC45232.1 (*Bacillus* sp. INT005). These structures were shown in Figure 2. The structure of PhaCs contained a canonical α/β hydrolase fold, consisting of a α/β core subdomain (the core catalytic subdomain) and a cap subdomain. The core catalytic subdomain was composed of 8 β-sheets surrounded by α-helices (Figures 2A–D). The cap subdomain of these enzymes all contained 6 α-helices, while Class I enzymes contained two reverse parallel β-sheets together with 6 α-helices (Figures 2A–D). There was also other structural divergence among these enzymes. For example, the 5th and 6th β-sheets of the core catalytic subdomain of Class I enzymes showed varied bending radian with the respective β-sheets of other enzymes (Figure 2E). The α-helices of the cap subdomain belonged to Class IV were shorter than those in the other Classes (Figure 2E). In addition, the cap subdomain had a short segment structure called lid region, which was highly dynamic and flexible. The change of the lid region was related to a closed or opened conformation of the enzyme, thus controlled the entrance of the substrate to the catalytic active pocket and affected the catalysis of PhaCs (Neoh et al., 2022).

**Figure 3:**
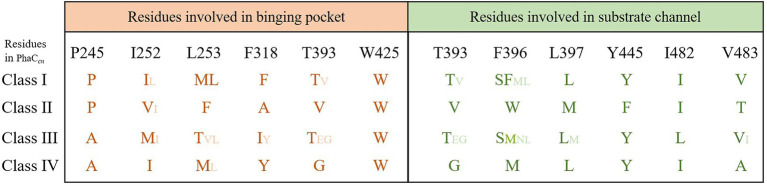
Residues involved in substrate binding pocket and substrate entrance channel of PHA synthases.

The size of substrate binding pockets and substrate-entrance channels might be the reason why PhaCs showed different substrate specificities. Studies found that P245, I252, L253, F318, T393 and W425 of PhaC from *C. necator* H16 formed a hydrophobic catalytic pocket, and these residues were relatively conservative in Class I, Class III, and Class IV PhaCs (Kim et al., 2017). Meanwhile, the substrate binding pocket of Class II PhaCs was composed of different residues (P, V, F, A, V and W), and they were highly conservative in Class II PhaC (Figure 3; Kim et al., 2017). At the conserved site (position 318 in Class I PhaC from *C. necator* H16), phenylalanine or tyrosine was predominant in class I, III and IV PhaCs, while the small side chain amino acid alanine was in class II PhaC (Figure 3), which might lead to larger substrate binding pockets and accommodation for larger substrates in class II PhaC. Additionally, T393, F396, L397, Y445, I482 and V483 constituted the substrate channel of Class I PhaC from *C. necator* H16 (Kim et al., 2017). Sequence alignment results showed that these amino acids were relatively conserved in class I, III and IV PhaCs, while V, W, M, F, I and T that constituted the substrate channel were highly conserved in class II PhaCs (Figure 3). These results indicated that substrate channels based on V, W, M, F, I and T might be more suitable for catalyzing the polymerization of substrates with long chains.

**Figure 4:**
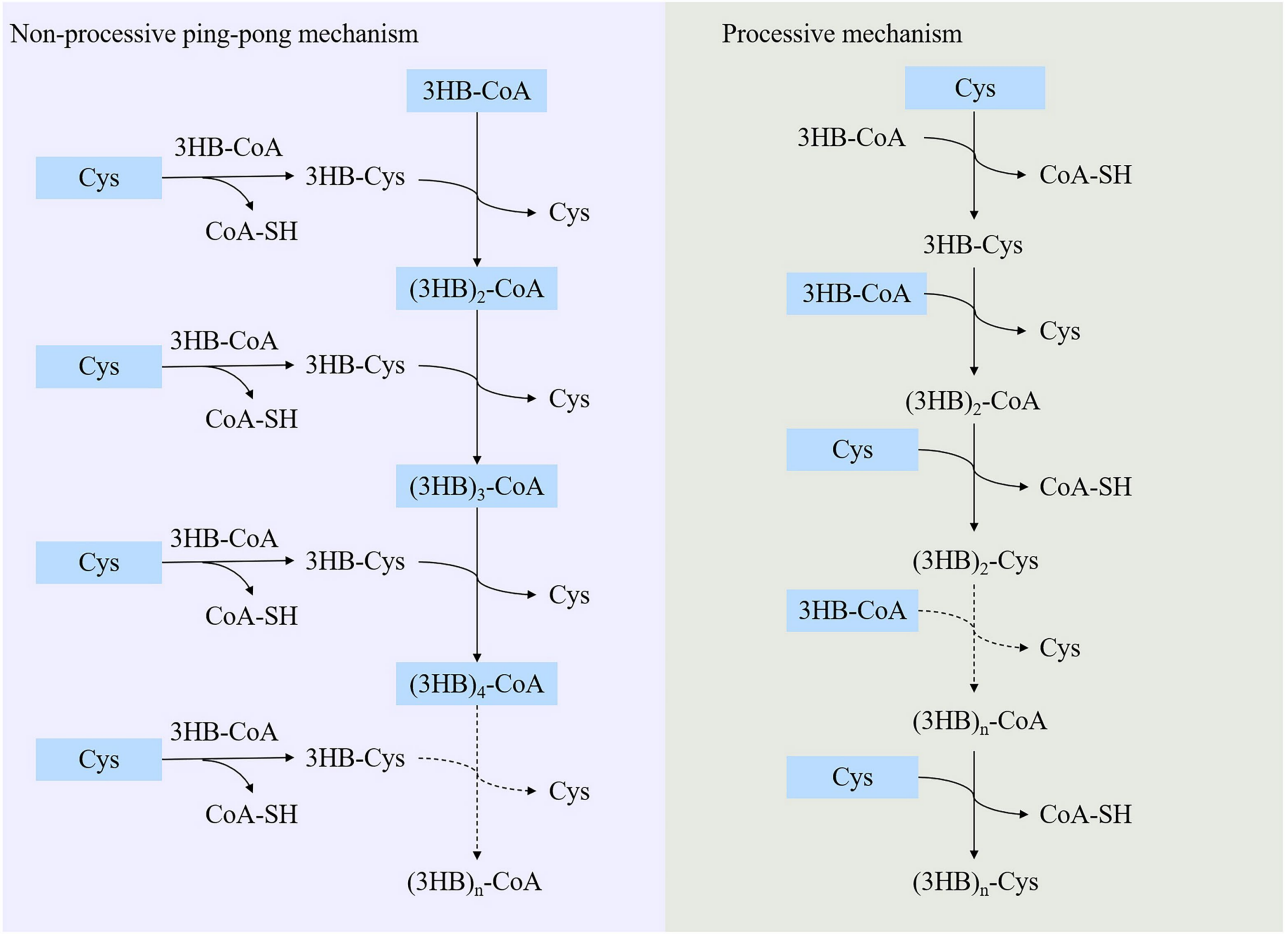
Catalytic mechanism of PHA synthase.

It was generally recognized that PhaC catalyzed PHAs synthesis in its dimeric form, and two catalytic mechanisms were proposed (Sagong et al., 2018). One was non-processive ping-pong model and the other was processive model (Sagong et al., 2018). The former model indicated that two active sites of the dimeric form PhaC were required to complete the elongation step. The substrate chain elongation of PHAs happened between the two cysteine residues in two protomer across the dimer interface, and the substrates and products shared the same channel during the catalytic process. Nevertheless, only one single catalytic active site was involved in completing the chain elongation in processive model. Two catalytic mechanisms were compared in detail (Sagong et al., 2018; Neoh et al., 2022). In general, cysteine nucleophilically attacked the thioester bond of acyl-CoA (such as 3HB-CoA), released CoA-SH and formed cysteine-3HB intermediate during the initial step. In the elongation step, the terminal hydroxyl group of substrate 3HB-CoA or (3HB)_n-1_-CoA was deprotonated and activated by histidine or aspartic acid, and then the activated substrate nucleophilic attacked thioester bond of 3HB-cysteine or (3HB)_n-1_-cysteine to generate (3HB)_n_-CoA (*n* ≥ 2; Figure 4).

### Sequences, structures and properties of enzymes for synthesizing PLA, PLGA, PCL, PBS, PEF and PES

As shown in Table 2, the enzymes that catalyzed the synthesis of PLA, poly(lactic-co-glycolic acid) (PLGA), PCL, PBS, PEF and PES were mainly lipase, and most of them were commercially available enzymes. In addition, some literatures referred to non-commercial ones such as LA polymerization enzyme (LPE), and carboxylesterase (Ma et al., 2009; Song et al., 2012). Among these enzymes, CALB was able to catalyze the synthesis of PLA, PLGA, PCL, PBS, PEF and PES, and exhibited excellent polymerization activity toward various substrates (Table 2). For example, PLGA was synthesized by using commercial CALB (Chanfreau et al., 2010), and a series of furanic–aliphatic polyesters such as PEF, poly(butylene furanoate) (PBF) and poly(hexamethylene furanoate) (PHF) could also be obtained *via* the CALB-catalyzed polymerization (Jiang et al., 2015; Nasr et al., 2022). However, studies showed that CALB exhibited higher polymerization efficiency toward lactones rather than lactide (Matsumoto and Taguchi, 2010). For example, the presence of lactide significantly reduced the rate of ε-caprolactone polymerization using CALB as the catalyst (Matsumoto and Taguchi, 2010). The polymerization temperature of these polyesters catalyzed by these enzymes was usually at 50°C - 90°C with toluene as the solvent except PES with the polymerization temperature at 125°C (Morales-Huerta et al., 2017). However, the performance of enzymes catalyzing polymers might be influenced by the solvent. For example, cutinase from *Humicola insolens* catalyzing PCL synthesis were investigated using different solvent systems (Hunsen et al., 2007). Results showed that the molecular weight increased from 16,000 to 24,900 Da by performing poly(ε-caprolactone) in toluene rather than in bulk (Hunsen et al., 2007).

**Figure 5:**
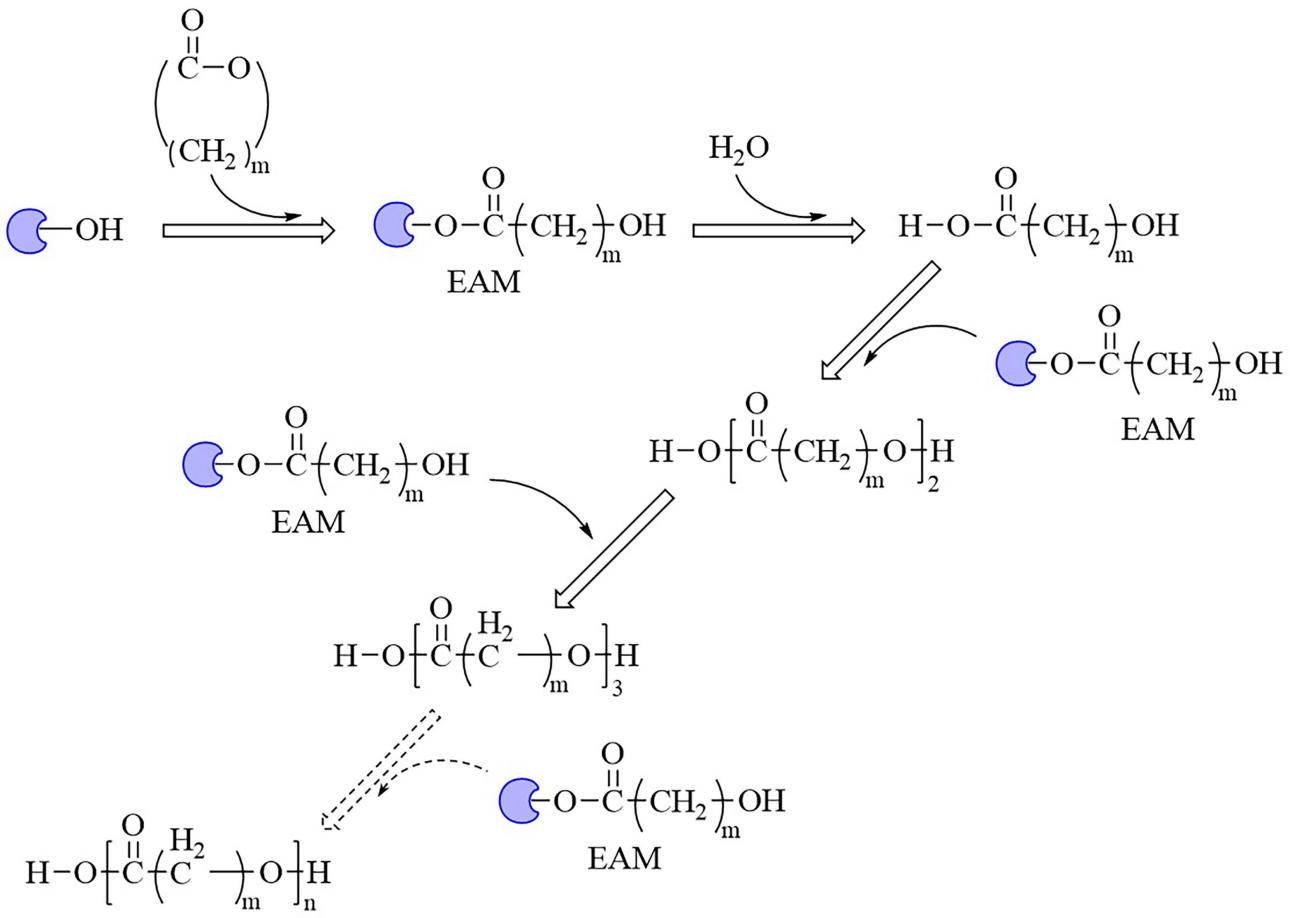
Mechanism of enzyme-catalyzed polymerization of lactones into polymers. 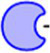, Enzymes; EAM, enzyme-activated monomer.

The properties of an enzyme depended on its sequence and structure. The sequence identities of these enzymes were all less than 28%. S-H-D were used as the catalytic triad for all enzymes except LPE (C-H-D), and all enzymes had G/A-X-S/C-X-G conserved sequence except CALB (T-X-S-X-G; Supplementary Figure S1). These enzymes also showed varied structures, for example, all enzymes harbored a reverse parallel arrangement of the second β-sheet except cutinase from *H. indolens* and lipase from *Fervidobacterium nodosum* Rt17-B1 with parallel arrangement in the same direction of β-sheets (Supplementary Figure S2). These enzymes also varied in the lid region that affected the substrate entrance into the catalytic active pocket. Although some kinds of enzymes such as cutinases carried no typical lid region, most of α/β hydrolases had a lid region, and this structure played an important role in substrate selectivity and catalysis (Kold et al., 2014). The mechanism of polyesters synthesis catalyzed by these above enzymes were relatively well studied. Taking lactones as an example, the polymerization mechanism of polyesters with enzymes as the catalyst were demonstrated as follows. The opening of the lid region allowed the lactones entering into the catalytic active pocket, and formed the enzyme-substrate complex, followed by the ring opening of the substrate to form an active acyl-enzyme intermediate (enzyme-activated monomer, EAM). Thereafter, nucleophilic reagents (such as water molecules) attacked the acyl-carbon of the EAM to form ester bonds and initiated polymerization. Next, the hydroxyl oxygen atom of the ring opening product attacked the acyl carbon atom of another EAM, and esterification reaction occurred and promoted the polymerization (Figure 5; Kobayashi, 2006). Some bio-plastics (e.g., PBAT and PGA) were yet not reported through enzymatic synthesis, although their monomeric structures harbored carboxyl and hydroxyl groups and were similar to those of enzymatically synthesized polyesters (Table 1). This might be due to the low catalytic efficiency of the enzymes or limited enzyme resources. One of the key limitations for bio-plastics in industrial applications was the synthetic efficiency, which was directly related to the price competitiveness of products. Therefore, the systematic review and comparison of the sequence, structure and catalytic mechanism of enzymes were not only contributed to clarify the catalytic properties of different enzymes, but also provided a theoretical basis for discovering and designing enzymes. The development of new enzymatic resources with higher catalytic efficiency and expanded application scope through rational or *de novo* protein design methods to improve production efficiency and reduce costs should be an important direction of enzymatic synthesis of bio-plastic polymers.

## Enzyme catalyzes the ester bond hydrolysis of plastics and PAEs

### Sequences, structures and properties of enzymes for PHAs and PLA hydrolysis

As one of the largest groups of bio-polyesters, PHAs received much attention as biodegradable plastics. Enzymatic ester-bond hydrolysis was the key step for the cleavage of the backbone of PHAs (Tarazona et al., 2020). Studies reported many PHAs- depolymerase and new group enzymes with varying properties were frequently identified (Supplementary Figure S3). Many of these enzymes were from marine bacteria, such as scl-PHA depolymerase (GenBank accession number BAA22882.1) from *Comamonas testosteroni* YM1004 isolated from seawater, and scl-PHA depolymerase (GenBank accession number BAC15574.1) from *Marinobacter* sp. NK-1 isolated from deep sea floor (Shinomiya et al., 1997; Kasuya et al., 2003). Compost was another main source for identifying degrading strains and related enzymes as the temperature was relatively high and suitable for thermotolerant species, and a temperature near or higher than the softening temperature of PHAs polymers was beneficial to the enzymatic hydrolysis of ester bond (Scherer et al., 1999; Takaku et al., 2006). There were also some enzymes from fungi, such as scl-PHA depolymerase from *Aspergillus fumigatus* (Jung et al., 2018). Compared with enzymes from bacteria, enzymes from fungi were relative rare (Supplementary Figure S3a).

According to the hydrolytic properties, these enzymes were mainly divided into two groups, mcl-PHA depolymerases and scl-PHA depolymerases (Supplementary Figure S3a). Polygenetic analysis indicated the mcl-PHA depolymerases and scl-PHA depolymerases each were further divided into two subgroups (Supplementary Figure S3a). The sequence alignment indicated that these four groups of enzymes showed distinctive conserved regions. Group 1 mcl-PHA depolymerases harbored conserve five peptides GHSQG (Supplementary Figure S3b), group 2 mcl-PHA depolymerases carried the conserved peptide sequence “GISSG” (Supplementary Figure S3c), and groups 1 and 2 scl-PHA depolymerases both showed the conserved region “GLSA/SG,” while at the forth position, the amino acid residue “S” was more frequently appeared in group 2 scl-PHA depolymerases (Supplementary Figures S3d,e). This might serve as the sequence characteristics when distinguishing different groups of PHA depolymerases. There were also some scl-PHA depolymerases not belonged to the two subgroups, indicating diverged sequence identities and possibly different catalytic properties (Supplementary Figure S3a). The sequence divergences of these enzymes might finally affect the protein structure and the catalytic mechanisms, including substrate specificity, the rates of hydrolysis and types of products.

Few studies focused on catalytic mechanisms of PHA depolymerases, and the structures of such enzymes were only PHA depolymerase from *Penicillium funiculosum* (PDB ID 2D81) and PHA depolymerase from *Paucimonas lemoignei* (PDB ID 4BRS). Both of them belonged to the serine hydrolase. The structure of PHB depolymerase from *Pen. funiculosum* was investigated together with the S39A mutant complexed with the methyl ester of a trimer (*R*)-3-PHB substrate. The catalytic triad S39-D121-H155 were located at topologically conserved positions. S40 and C250 of the main chain amide groups formed the oxyanion hole (Hisano et al., 2006). A crevice was located on the surface of the PHA depolymerase and benefited for substrate binding through hydrophobic interactions with several hydrophobic residues. Thirteen hydrophobic residues were located around the crevice, and contributed to the binding affinity of the enzyme to PHB substrates. The structure of the mutant S39A–trimeric substrate complex showed the residue W307 was responsible for ester bond recognition, and at least three subsites were included for binding monomer units of substrates (Hisano et al., 2006). Structure analysis of *Pau. lemoignei* depolymerase provided us a more precise insight into substrate binding and catalysis (Jendrossek et al., 2013). Structural analysis of PHB depolymerase PhaZ7 (S136A) with 3-hydroxybutyrate tetramer complex indicated the substrate binding cleft was composed of Y105, Y176, Y189 and Y190, and site-directed mutagenesis of these five sites confirmed their importance. All these residues were located at a single surface-exposed location (Jendrossek et al., 2013). After substrate binding, the catalytic active residue S136 nucleophilically attached the substrate and started hydrolysis. The proposed activation mechanism involved a conformational change of the lid region (281–294 loop) leading to the opening of the active-site entrance channel and interaction with the hydrophobic substrate (Jendrossek et al., 2013). Studies found that *A. fumigatus* depolymerase showed the properties of both endo- and exo-modes of hydrolysis to degrade PHB and 3HB oligomers, indicating endo- and exo-cleavage mechanisms of PHA depolymerases but still needed in-depth investigation (Scherer et al., 2000).

Besides PHAs, PLA was another kind of bio-plastic widely used. Enzymatic depolymerization of PLA began in the 1980s (Williams, 1981). Studies continuously focused on enzymatic PLA hydrolysis since then, and a series of enzymes were identified, including protease, lipase, cutinase, esterase and PLA depolymerase mainly from bacteria, such as *Actinomadura keratinilytica*, *Amycolatopsis orientalis*, *Kibdelosporangium aridum*, *Laceyella sacchari*, *Pseudonocardia* sp., and *Streptomyces roseolus*. Studies also reported other sources for PLA depolymerases such as the enzymes from fungi (Zaaba and Jaafar, 2020). The catalytic mechanisms of related enzymes were deeply analyzed. For example, the PLA degradation mechanism of proteases K indicated that the catalytic process took place in four stages, including substrate binding, nucleophilic attack, protonation, and ester bond hydrolysis, and catalytic active residue S329 attacked the ester bonds of the substrate as the nucleophile (Xiang et al., 2017). Based on the ester bond hydrolysis characteristics of related enzymes, the degrading and recycling of PLA were realized, so this part will not repeatedly summarize related enzymes and the catalytic mechanism. PLA materials also showed disadvantages, such as poor hydrophilicity, wide range of molecular weight distribution, weak strength, and poor processing heat resistance, thus limited PLA manufacturing (Bai et al., 2017; Tawiah et al., 2018). Therefore, some studies developed co-polymerized lactic acid molecules with other monomers to produce derivative polymers, and one of the representatives was poly(lactide-co-glycolide) (PLGA). PLGA hydrolysis by commercial hydrolase were performed, and among three kinds of commercially available hydrolase esterases, lipases and proteases, the lipases from *C. antarctica*, *C. cylindracea*, *C. rugosa*, *Mucor miehei*, *Rhizopus arrhizus* and porcine pancreas and the esterase from *M. miehei* showed good performance for PLGA degradation (Kemme et al., 2011). However, due to the limited substrate spectrum of the enzyme, the hydrolysis mechanisms of the specific degrading enzymes for PLA derivatives were still not yet studied thoroughly.

### Sequences, structures and properties of enzymes for PCL, PBS and derivatives, and PES hydrolysis

**Figure 6:**
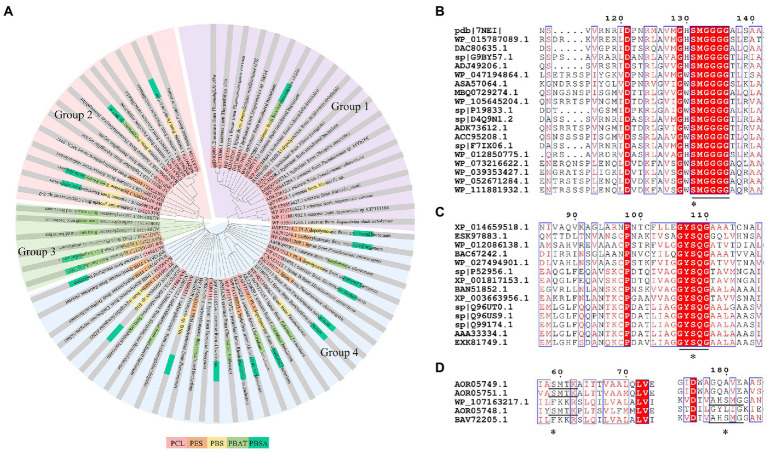
Sequence alignment of enzymes catalyzing the hydrolysis of PCL, PES, PBS, PBSA, and PBAT. **(A)** The phylogenetic tree of PCL, PES, PBS, PBSA, and PBAT depolymerase. The fan with light purple represents group1; the fan with light red represents group2; the fan with light green represents group3; the fan with light bule represents group4. **(B)** Sequence alignment of Group 1 enzymes, **(C)** Sequence alignment of Group 2 enzymes. **(D)** Sequence alignment of Group 3 enzymes. “*” The catalytic active site.

Enzymes related to the degradation of PCL, PBS and derivatives, and PES were all α/β fold hydrolases, mainly including lipase, cutinase and carboxylesterase (Maeda et al., 2005; Almeida et al., 2019; Rosato et al., 2022). The phylogenetic relationship analysis based on enzymatic sequences was performed of these enzymes (Figure 6A). These enzymes could be divided into four groups according to the characteristics of their respective substrates. Enzymes degrading PCL were mainly belonged to group 1 and group 2. Enzymes in group 1 showed highly conserved GH/WSMGGGG sequences (Figure 6B). Enzymes belonged to group 2 showed the highly conserved GYSQG sequence (Figure 6C). Few studies focused on the crystal structures of PCL hydrolases, and only two enzymes were identified in the PDB database, including carboxylesterase (MGS0156) from uncultured organism (PDB ID 5D8M) and esterase (AfEST) from *Archaeoglobus fulgidus* (PDB ID 1JJI). The structure of MGS0156 was composed of the α/β hydrolase fold together with a lid region. As the enzyme showed relatively strong binding affinity with long chain oligomers, short oligomers were generated one by one under enzymatic catalysis without repeatable binding and hydrolysis (Feng et al., 2020). The molecular mechanism of enzymatic hydrolysis of PCL was investigated. This process was mainly divided into two stages, acylation (triad-assisted nucleophilically attacked to the substrate) and hydrolysis (C-O bond cleavage of the substrate), and the second stage was recognized as the rate-determining step (Feng et al., 2020). Additionally, studies indicated that G168, H231, D237, D372 and K382 residues played an important role during PCL hydrolysis (Feng et al., 2020). AfEST also harbored the classic α/β hydrolase domain and a cap domain. The catalytic mechanism of AfEST was similar to that of MGS0156. First, a nucleophilic attack performed by the catalytic S160 residue, and G88, G89 and A161 formed the oxyanion hole to stabilize the intermediate. Then the C − O bond was cleaved to form 6-hydroxycaproic acid (monomer) and acyl−enzyme intermediate. Thereafter, acyl−enzyme intermediate was deacylated with one water molecule attack and released the product (Almeida et al., 2019).

PBS was industrialization for many years due to their thermo-mechanical and physical properties since the first commercialized PBS produced by Showa High polymer (Xu and Guo, 2010). By copolymerization with other dicarboxylic acids or diols, PBS derivatives (such as PBSA and PBAT) also came into plastic market (Xu and Guo, 2010). As shown in Figure 6A, PBS derivatives related degrading enzymes mainly belonged to group 3 and group 4. Group 3 mainly included the enzymes degrading PBAT. Sequence alignment analysis showed that these enzymes contained the common catalytic residue serine, while showed different conserved residues (SMTK or AHSM) near the catalytic active site serine (Figure 6D), indicating that there might be divergences in catalytic properties of these enzymes. Many of the enzymes in group 4 showed the ability to degrade multi-kinds of PBS derivatives (Figure 6A). Studies found that enzymes showed varied substrate preferences when catalyzing different kinds of PBS polyesters, mainly affected by the carbon chain length of the monomer carboxylic acid of the substrate (Maeda et al., 2005). For example, the cutinase from *A. oryzae* showed a preference toward PBSA rather than PBS (Maeda et al., 2005). Similarly, the cutinase from *Pseudozyma antarctica* JCM 10317 showed a higher degrading rate toward PBSA than that of PBS (Shinozaki et al., 2013). However, the enzymatic degradation mechanisms were not yet in-depth investigated for PBS derivatives, and they mainly focused on the hydrolysis mode, including endo-type hydrolysis and exo-type hydrolysis. Studies found that the catalytic process of cutinase hydrolyzing PBS could be explained by endo-type mechanism, meanwhile, the cutinase hydrolyzed PBSA through the exo-type mode (Rosato et al., 2022), which might be related to the hydrolysis preferentially occurring at the ester groups with much more methylene contents (Bai et al., 2018). In addition, there were PES degrading enzymes in group 4 (Figure 6A), mainly PHB and PLA depolymerases, such as P(3HB) depolymerase from *A. clavatus* NKCM 1003 (Ishii et al., 2007), and PHB depolymerase from *A. fumigatus* (Jung et al., 2018). However, studies focused on PES degrading enzymes were rare and hardly any studies about the enzymatic degradation mechanism of PES was reported.

### Sequences, structures, and properties of enzymes for PEF hydrolysis

Although PEF polymers were considered to be biodegradable, there were few studies about enzymatic PEF degradation, and only three enzymes were reported with the ability to degrade PEFs, including cutinase from *Thermobifida cellulosic*, cutinase from *H. insolens* (HiC), and PETase from *Ideonella sakaiensis* 201-F6. (Pellis et al., 2016) used the cutinase from *T. cellosilica* to firstly investigate the enzymatic degradation on PEF, and found that the enzyme preferentially hydrolyzed PEF with higher molecular weights, and produced 2,5-furandicarboxylic acid and oligomers. Other studies used enzyme HiC to degrade PEF films and a complete hydrolysis of PEF films was achieved within 72 h (Weinberger et al., 2017). Recently, the PETase from *I. sakaiensis* 201-F6 was found to hydrolyze PEF, and the hydrolysis activity of PETase was enhanced after site directed mutation (S238F/W159H; Austin et al., 2018). As the industrialization of PEF was in infancy, few studies focused on the catalytic mechanisms of PEF by enzymes, and thus would be an important direction in the future.

In short, many studies on the depolymerization of the above bio-plastics were based on the structure characteristic of such polymer materials connected by ester bonds, thus were recognized as easy to be degraded. Some of the degradation studies only stayed on the phenomenon of microbial depolymerization, and did not deeply investigated the sequence and structural characteristics of depolymerases, and yet did not well revealed the molecular mechanism of ester bond cleavage of those enzymes. Although the bio-plastics could be biodegraded, only relying on the natural degradation still took a long half-life to depolymerize, such as PLA (Rosli et al., 2021). How to efficiently realize hydrolysis and recycling of related bio-plastics is still a mainstream and hot topic in future studies. Mining and rational design of enzymes will certainly provide effective tools for efficient depolymerization and recycling of these bio-polyesters.

### Sequences, structures, and properties of enzymes for PET hydrolysis

**Figure 7:**
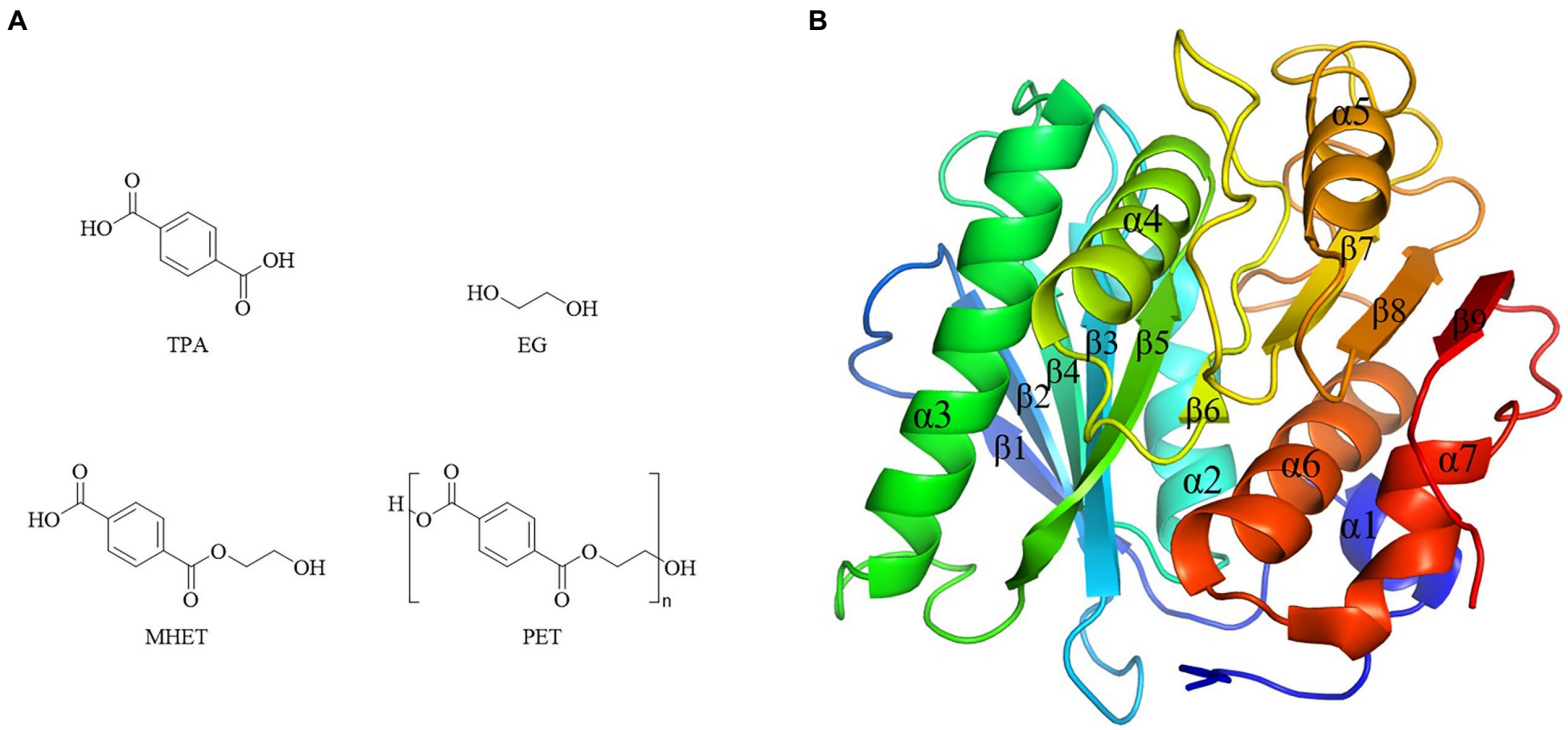
Chemical structure of PET and monomers, and PETase structure. **(A)** Chemical structure of PET and monomers. **(B)** PETase structures (PDB ID 5XJH).

Bio-plastics showed short degradation half-life in natural environment compared with petro-plastics, thus drew great attentions by researchers. However, the dominating plastics were still petro-plastic products nowadays, typically represented by five kinds of most commonly used petro-plastics PE, PP, PVC, PS and PET, and among them PET was the only polymers contained ester-bond in the backbone (Verschoor et al., 2022). PET showed the advantages of light weight, insulation and durability, and widely used to produce synthetic fibers, as well as containers or outer packages of various liquids and solids in foods, cosmetics and daily chemical products industries (Kawai et al., 2020). The production of PET in 2021 was about 27.45 million tons, and the demands will be greatly increased in the future as the recycling property of PET (Kawai et al., 2020). PET was composed of ester bond-linked terephthalate (TPA) and ethylene glycol (EG) (Figure 7A). It had the hydrolysable ester bonds, but very stably in the natural environment, which was difficult to degrade with a half-life of hundreds of years. Enzymes were the preferred choice to treat with PET waste. Enzymatic ester bonds hydrolysis of PET made great progress in recent years.

The first report of enzymatic PET hydrolysis dated back to 2005 (Müller et al., 2005), and it found that the polyester hydrolase from *T. fusca* DSM43793 (TfH) could hydrolyze about 40–50% PET films in three weeks, and since then PET hydrolases attracted much attention. A series of enzymes capable of hydrolyzing PET were identified, mainly carboxylic ester hydrolases including lipases, carboxylesterases, cutinases, and PETase (Gao et al., 2021). Among them, PETase from *I. sakaiensis* showed the high hydrolytic efficiency of PET at a relative low temperature (Yoshida et al., 2016). For example, its hydrolytic activity on PET at 30°C – 40°C was significantly higher than the previously reported enzymes PET hydrolases *Tf* HCut (*T. fusca* cutinase), LCC (leaf-branch compost cutinase) and FsC (*Fusarium solani* cutinase) (Yoshida et al., 2016). Due to the high catalytic efficiency toward PET, the crystal structure of PETase was extensively studied. There were 40 crystal structures of PETases in the PDB database, including protein structure, protein substrate complex, and mutanted PETases (Joo et al., 2018). PETase had the typical α/β hydrolase fold structure, with the core domain consisting of 9 β-sheets surrounded by 7 α-helices, and the catalytic triad of S160-D206-H237 (Figure 7B). Compared with other PET hydrolases, PETase had three extra residues (N244, S245, N246) in the β8-α6, which extended the cleft of the substrate binding pocket and enlarged the binding pocket toward macromolecular substrate (Joo et al., 2018).

According to the crystal structure, the catalytic mechanism of PETase was revealed through molecular docking, site directed mutagenesis and enzymatic property analysis (Han et al., 2017; Joo et al., 2018). The hydrolysis of PET by PETase included two steps, nick generation step and terminal digestion step. In the first step, PET combined to the substrate binding pocket on the surface of enzyme through hydrophobic interaction. Specifically, one mono(ethylene terephthalate) (MHET) was bound to subsite I and three MHETs to subsite II, and thus a long and shallow L-shaped cleft was formed on a flat surface. Thereafter, S160 nucleophilically attacked the carbonyl carbon atom in the ester bond between subsite I and subsite II, generating the PET chain with hydroxyethyl-terminal (HE-terminal) and acyl-enzyme intermediates. Subsequently, acyl-enzyme intermediate was nucleophilically attacked by the hydroxyl oxygen atom of a water molecule, generating PET chains with TPA terminal of which the benzene ring was interacted by the benzene ring of W156 with π stacking interaction rather than original weaker T-stacking. Finally, the product was rotated and pulled away from original position before released. The second step was the terminal digestion step. PET chains with TPA terminal or HE-terminal continued binding to the subsite, and finally generated MHET, TPA and EG through the nick generation step.

In recent years, the hydrolysis of intermediates MHET (Figure 7A) was also well investigated. Studies indicated that MHETase from *I. sakaiensis* could hydrolyze MHET to TPA and EG (Yoshida et al., 2016). In addition, the MHETase showed a very narrow substrate specificity, which only hydrolyzed MHET, but could not hydrolyze BHET, PET, *p*-nitrophenyl aliphatic esters or aromatic ester compounds (Yoshida et al., 2016). Nowadays, there were 10 crystal structures of MHETases in the PDB database, mainly including crystal structure of MHETase bound to ligand, and MHETase at different resolutions (Palm et al., 2019). The overall structure of MHETase was similar to that of feruloyl esters (Palm et al., 2019), with α/β hydrolase domain, lid region (a α-helix), and catalytic triad S225-D492-H528 (Knott et al., 2020). By analyzing the crystal structure of MHETase and nonhydrolyzable MHETA (mono-(2hydroxyethyl) terephthalamide), (Palm et al., 2019) found that the substrate specificity of MHETase was almost completely determined by the lid region. Furthermore, through the combination of PETase and MHETase, ester bonds of PET could be completely hydrolyzed to produce the monomers TPA and EG, indicating a promising two-enzyme system for recycling of PET (Palm et al., 2019).

### Sequences, structures, and properties of enzymes for PAEs hydrolysis

As a typical representative of plastics, the degradation of PET became a hot topic in recent years. However, it was easy to ignore that for the plastic industry, plasticizers were another kind of pollutants urgently needed to be treated. PAEs, the representative of plasticizers were di-ester chemicals formed by esterifying the hydroxyl group of alcohols with the carboxyl group of phthalic acid (Tian et al., 2019). Studies showed that the hydrolysis of the di-ester bonds of PAEs was step by step (Zhang et al., 2014). Meanwhile, the toxicity of PAEs also depended on the side chain ester bond structures, and the hydrolysis of one ester bond of PAEs could significantly reduce its toxicity (Jiao et al., 2013; Tian et al., 2019). For example, dimethyl phthalate and diethyl phthalate were associated with androgenic effects, and the corresponding phthalate mono-ester lost relevant function, meanwhile dibutyl phthalate and di(2-ethylhexyl) phthalate caused steroid hormone effects, and the effects caused by related phthalate mono-esters were significantly reduced (Tian et al., 2019). Therefore, identifying PAEs side chain ester bond hydrolases could provide optimal enzyme resources for the reduction or elimination of PAEs toxicity (Tian et al., 2019).

Studies about side chain ester bond hydrolase of PAEs mainly included lipase, esterase, cutinase and phthalate ester hydrolase in recent 15 years (Table 3). These enzymes had different substrate specificity when hydrolyzing side chain ester bonds, and could not effectively hydrolyze different kinds of side chain ester bonds of PAEs (Table 3). According to the hydrolysis characteristics of side chain ester bonds, these enzymes could be divided into three categories, type I, II and III. Type I enzymes hydrolyzed only one ester bond of PAEs. Type II enzymes hydrolyzed the only side chain ester bond of phthalate mono-ester, and type III enzymes could hydrolyze the two ester bonds of PAEs. Previous studies investigated the hydrolysis of the di-ester bonds of PAEs with the combination of type I and II enzymes (Huang et al., 2019). However, compared with the combination of type I and II enzymes, the type III enzymes were more economical in protein purification and practical application, because the expression and purification of type I and II enzymes brought more expenditure and operational complexity, and the optimal reaction conditions of different enzymes were also diverged. Therefore, type III enzymes became the preferred enzyme resource for thoroughly hydrolyzing the di-ester bond of PAEs. However, there were few type III enzymes until now, among which *F. oxysporum* cutinase were only reported with the ability to degrade di-pentyl phthalate (Ahn et al., 2006). The carboxylesterase from *Bacillus* sp. K91 only showed the ability to degrade di-isobutyl phthalate (Ding et al., 2015), and two soil derived esterases also showed obvious substrate specificity, preferring to hydrolyze the ester bonds when linked with short straight chain of PAEs (Wu et al., 2019; Qiu et al., 2020).

**Table 3:** A summary of enzymes for ester bond hydrolysis of phthalate esters derived from microorganisms.

Name	Classification^a^	Substrate spectrum	Species	Reference
Esterase	I	Di-pentyl phthalate.	*Candida cylindracea*	Ahn et al. (2006)
Esterase	I	Di-methyl phthalate.	*Fusarium* sp. DMT-5-3	Luo et al. (2012)
Phthalate hydrolase	I	Di-propyl phthalate, di-butyl phthalate and di-pentyl phthalate.	Metagenomic Library	Jiao et al. (2013)
Phthalate hydrolase	I	Di-methyl phthalate, di-ethyl phthalate, di-propyl phthalate, di-butyl phthalate, di-pentyl phthalate and di-hexyl phthalate.	*Acinetobacter* sp. M673	Wu et al. (2013)
Esterase	I	Di-ethyl phthalate, di-propyl phthalate, di-butyl phthalate, di-pentyl phthalate, di-hexyl phthalate and butylbenzyl phthalate.	*Sulfobacillus acidophilus* DSM10332	Zhang et al. (2014)
Esterase B and Esterase G	I	Di-butyl phthalate. Esterase G was more efficient for di-butyl phthalate hydrolysis compared with that of Esterase B.	*Sphingobium* sp. SM42	Whangsuk et al. (2015)
Esterase	I	Di-ethyl phthalate, di-propyl phthalate, di-butyl phthalate, di-pentyl phthalate and di-hexyl phthalate.	*Sphingomonas glacialis* PAMC 26605	Hong et al. (2016)
Esterase	I	Di-butyl phthalate.	*Acinetobacter* sp. LMB-5	Fang et al. (2017)
Lipase	I	Di-methyl phthalate, di-ethyl phthalate, di-propyl phthalate, di-butyl phthalate, di-isobutyl phthalate, di-hexyl phthalate and di-(2-ethylhexyl) phthalate. The enzyme showed a relatively high efficiency for hydrolysis of di-propyl phthalate, di-butyl phthalate and di-hexyl phthalate.	*Malbranchea cinnamomea* CGMCC 6022	Duan et al. (2019b)
Cutinase	I	Di-methyl phthalate, di-ethyl phthalate, di-propyl phthalate, di-butyl phthalate, di-isobutyl phthalate, di-hexyl phthalate. The enzyme showed a relatively high efficiency for hydrolysis of di-methyl phthalate, di-ethyl phthalate, di-propyl phthalate and di-butyl phthalate.	*Thielavia terrestris*	Duan et al. (2019a)
Esterase	I	Di-methyl phthalate, di-ethyl phthalate, di-propyl phthalate, di-butyl phthalate, di-isobutyl phthalate, butylbenzyl phthalate, di-pentyl phthalate, di-hexyl phthalate, di-n-octyl phthalate, di-(2-ethylhexyl) phthalate and di-cyclohexyl phthalate. The enzyme showed a relatively high efficiency for hydrolysis of di-methyl phthalate, di-ethyl phthalate, di-propyl phthalate, di-butyl phthalate, di-isobutyl phthalate and butylbenzyl phthalate.	*Gordonia* sp. 5F	Huang et al. (2019)
Carboxylesterase	I	Di-butyl phthalate.	*Sphingobium yanoikuyae* P4	Mahajan et al. (2019)
Carboxylesterase	I	Di-methyl phthalate, di-ethyl phthalate, di-propyl phthalate and di-butyl phthalate. The preferred substrate was di-butyl phthalate.	*Bacillus velezensis* SYBC H47	Huang et al. (2020)
Esterase	I	Di-butyl phthalate. The *K*_m_ and *k*_cat_ values for di-butyl phthalate were 9.60 ± 0.97 μM and (2.72 ± 0.06) × 10^6^ s^−1^, respectively.	*Microbacterium* sp. PAE-1	Lu et al. (2020)
Serine hydrolase	I	Di-butyl phthalate, di-isobutyl phthalate, and di-(2-ethylhexyl) phthalate.	*Bacillus subtilis* BJQ0005	Xu et al. (2021b)
Alpha/beta fold hydrolase	I	Di-(2-ethylhexyl) phthalate.	*Bacillus subtilis* BJQ0005	Xu et al. (2021b)
Phthalate hydrolase	II	Mono-ethyl phthalate, mono-butyl phthalate, mono-hexyl phthalate and mono-(2-ethylhexyl) phthalate.	*Gordonia* sp. P8219	Nishioka et al. (2006)
Phthalate hydrolase	II	Mono-methyl phthalate.	*Rhodococcus jostii* RHA1	Hara et al. (2010)
Phthalate hydrolase	II	Mono-(2-ethylhexyl) phthalate.	*Rhodococcus* sp. EG-5	Iwata et al. (2016)
Phthalate hydrolase	II	Mono-methyl phthalate, mono-ethyl phthalate, mono-butyl phthalate and mono-(2-ethylhexyl) phthalate.	*Gordonia alkanivorans* YC-RL2	Nahurira et al. (2017)
Phthalate hydrolase	II	Mono-(2-ethylhexyl) phthalate.	*Pseudomonas* sp.	Singh et al. (2017)
Phthalate hydrolase	II	Mono-ethyl phthalate, mono-butyl phthalate, mono-hexyl phthalate and mono-(2-ethylhexyl) phthalate.	*Gordonia* sp. YC-JH1	Fan et al. (2018)
Phthalate hydrolase	II	Mono-methyl phthalate, mono-ethyl phthalate, mono-propyl phthalate, mono-butyl phthalate, mono-isobutyl phthalate, mono-amyl phthalate, mono-hexyl phthalate, mono-n-octyl phthalate, mono-(2-ethylhexyl) phthalate and mono-cyclohexyl phthalate.	*Gordonia* sp. 5F	Huang et al. (2019)
Esterase	II	Mono-butyl phthalate.	*Microbacterium* sp. PAE-1	Lu et al. (2020)
Cutinase	III	Di-pentyl phthalate.	*Fusarium oxysporum*	Ahn et al. (2006)
Carboxylesterase	III	Di-isobutyl phthalate.	*Bacillus* sp. K91	Ding et al. (2015)
Feruloyl esterase	III	Di-methyl phthalate, di-ethyl phthalate and di-butyl phthalate. The preferred substrate was di-methyl phthalate.	Soil metagenomic library	Wu et al. (2019)
Phthalate hydrolase	III	Di-methyl phthalate, di-ethyl phthalate, di-propyl phthalate, di-butyl phthalate, di-pentyl phthalate and di-hexyl phthalate.	Soil metagenomic library	Qiu et al. (2020)
Carboxylesterase	III	Di-butyl phthalate, di-isobutyl phthalate and di-(2-ethylhexyl) phthalate.	*Bacillus subtilis* BJQ0005	Xu et al. (2021b)

^a^ I, Conversion of di-ester phthalate to mono-ester phthalate; II, Conversion of mono-ester phthalate to phthalic acid; III, Conversion of di-ester phthalate to phthalic acid.

In short, type III enzymes resources were relatively scarce. So far, there were only four known type III enzyme sequences obtained from literatures, of which feruloyl esterase and phthalate hydrolase were identified from the metagenomic library of soil, and the cutinase was from *F. oxysporum*. The left type III enzyme carboxylesterase was from *B. subtilis* BJQ0005 by our team (Xu et al., 2021b) and from *Bacillus* sp. K91 by Ding et al. (2015), and these two enzymes with the sequence identity of 100%. The type III enzyme resources were still unable to meet the requirements for various PAEs treatment conditions such as food industry and environmental systems with varied pH, temperatures and reaction solvent conditions. Clarifying the corresponding sequence and structural characteristics, and analyzing the catalytic mechanism of related enzymes were the possible way to change the status in view of the scarcity of type III enzyme resources and their limited substrate spectrum. Therefore, the sequences, structural characteristics and catalytic mechanisms of PAEs hydrolases reported so far were summarized.

**Figure 8:**
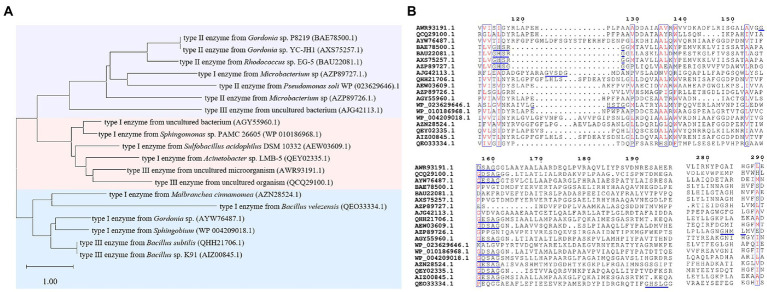
Sequence analysis of PAEs hydrolases. **(A)** The phylogenetic tree of PAEs hydrolases. **(B)** Sequence alignment of PAEs hydrolases, the blue line marks the conserved sequence of the enzyme.

**Figure 9:**
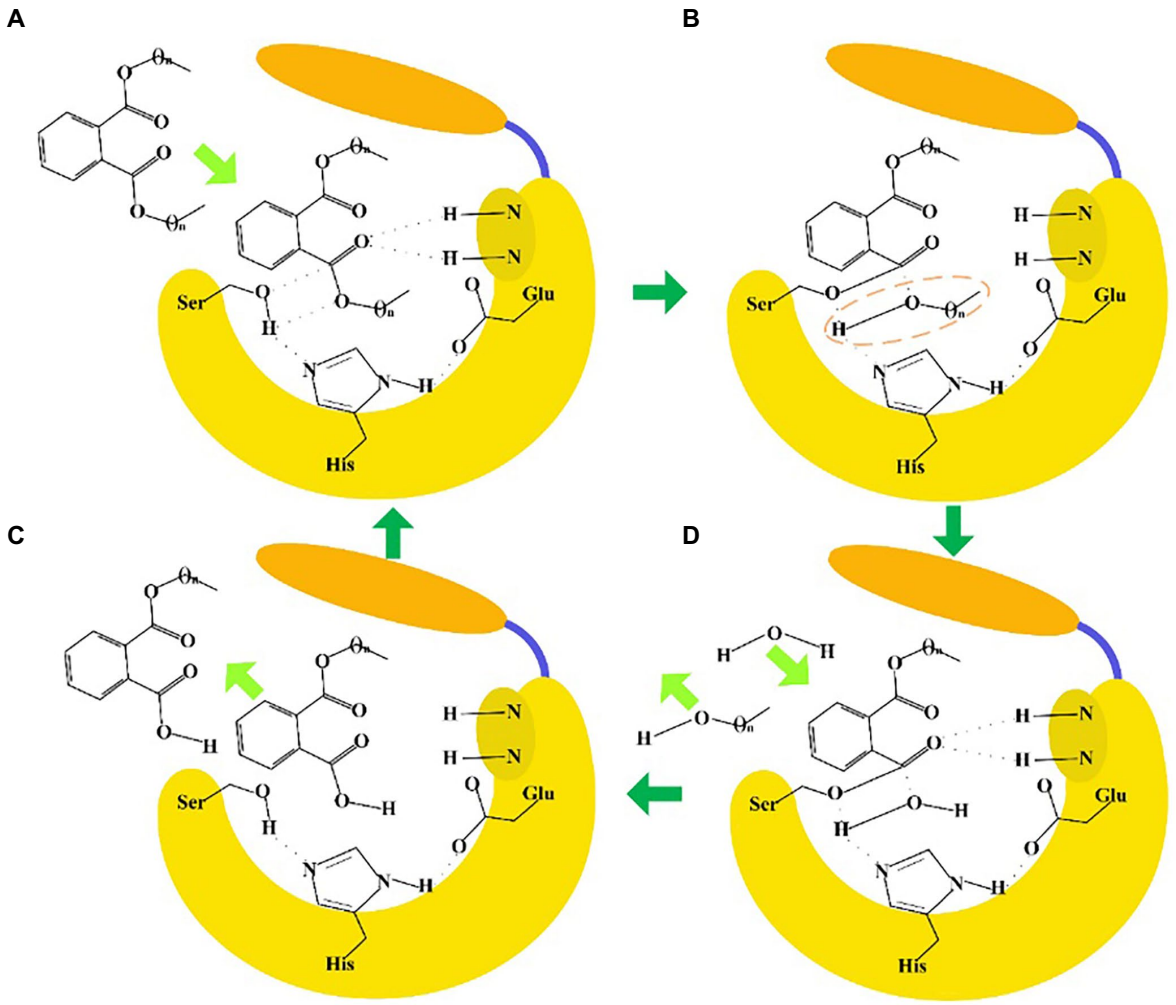
Enzyme-catalyzed ester bond hydrolysis of PAEs. **(A)** When the lid region opened, PAEs entered into the substrate binding pocket to form a tetrahedral transition state. **(B)** The Ser hydroxyl oxygen atom nucleophilic attacked the carboxyl carbon atom of the substrate to form a covalent bond, and the covalent bond was broken between the carboxyl carbon atom and the oxygen atom of the substrate. **(C)** One water molecule entered into the substrate binding pocket to form a tetrahedron transition state. **(D)** The carboxyl carbon atom of the substrate formed a covalent bond with the oxygen atom of water, and the covalent bond was broken between Ser hydroxyl oxygen atom with the carbon atom of the substrate. Finally, the product escaped from the catalytic pocket to complete the ester bond hydrolysis reaction.

The sequence identities of PAEs hydrolases were quite varied. Results of the phylogenetic tree showed that these enzymes were clustered into three major categories (Figure 8A). The first category was dominated by type II enzymes, and the second and third categories contained both type I and type III enzymes, which suggested that type I and type III enzymes showed a relatively closed relationship in sequence similarity, and could not be clearly distinguished from each other by sequence identity (Figure 8A). Meanwhile, S-H-D formed the catalytic triad of all the type I, II and III enzymes, except MpeH, a type II enzyme from the bacterial consortium. MpeH had a consensus motif GHML identified in some serine hydrolases, while the other enzymes had the typical G-X-S-X-G conserved sequence (Figure 8B). For type III enzymes, all of them had the conserved sequence G-E/D-SAGG, except EstM2 obtained from the metagenome (Figure 8B). For the representative ester bond hydrolases such as lipase, esterase and cutinase, the structural characteristic of enzymes and the PAEs hydrolysis mechanism were studied (Rajagopalan et al., 2014; Höllerer et al., 2018; Figure 9). These enzymes belonged to α/β hydrolase family, and generally carried a lid region composed of α-helices, and the α-helix connected with the core domain through hinge (usually a loop region). The lid region usually covered the catalytic active pocket, and the opening of the lid region allowed the substrate to enter into the substrate binding pocket. The catalytic triad was Ser-His-Glu (Asp), connected by hydrogen bonds, and histidine was polarized by the carboxyl group of glutamic acid (aspartic acid), and tended to receive protons from serine, and prompted the hydroxy oxygen atom of serine to nucleophilic attack the carboxyl carbon atom of the substrate (Rajagopalan et al., 2014; Höllerer et al., 2018). A pair of hydrogen atoms located in the 3 Å – 5 Å region around the serine hydroxyl group could be used as hydrogen bond donors to stabilize the tetrahedral transition state formed in the catalytic process, and was called as oxygen anion hole. The cavity between the lid region, the oxygen anion hole and the catalytic active center were the substrate binding pocket (Rajagopalan et al., 2014; Höllerer et al., 2018).

Although the enzymatic ester bond hydrolysis processes of PAEs were proposed, divergences in catalytic mechanism of different enzymes still required in-depth investigation. For example, in our previous work about ester bond hydrolysis of type III enzyme GTW28_17760, H399 of the catalytic active site was found to form a hydrogen bond interaction with the carboxyl group of the substrate mono-butyl phthalate, thus reducing the hydrolysis efficiency of the enzyme catalyzing the hydrolysis of the following ester bonds of the substrate (Xu et al., 2021b). How to overcome this catalytic issue became the key point to improve the catalytic efficiency. In addition, there were significant divergences in the sequences and structures of different enzymes, such as the enzymes belonged to type III enzyme, which indicated that there might be differential hydrolysis mechanisms for them to catalyze the di-ester bonds of PAEs and needed to be further investigated. Revealing the sequence characteristics and catalytic mechanisms of type III enzymes were helpful to guide the directional discovery and rational design of related enzymes, thus enriching the resources of type III enzymes and providing a basis for the effective establishment of enzymatic treatment methods for PAEs required by different reaction systems and conditions.

Based on the discussions in parts 3 and 4, it could be concluded that many enzymes belonged to the α/β hydrolase family played a crucial role in synthesis and hydrolysis of these polyesters (Supplementary Figure S4). Comparative discussion on the mechanisms of enzymatic ester bond catalysis is helpful to deeply reveal the synthesis and hydrolysis process of polymers from the perspective of enzymology, so as to better improve relevant processes, and better recycling of bio-plastics to reduce environmental pressure. In fact, enzymatic ester bond synthesis and hydrolysis could be applied in many aspects besides plastic industry, such as the traditional fermented food industry. Ester bond synthesis or hydrolysis by enzymes played a crucial role on formation of flavor esters and degradation of potential harmful esters, thus greatly improved product quality (Xu et al., 2020, 2021a, 2023; Zhao et al., 2023). In short, enzymatic ester bond synthesis and hydrolysis is a research hot topic worth continuous studies.

## Bottlenecks and challenges of recycling plastics

Plastics were indispensable products in modern society, but the acquisition of plastic monomers from fossil and the generation of plastic waste brought a serious threat to the global climate and ecological environment (Service, 2021). With respect to the treatment of plastic waste, recycling was a more promising method than the traditional landfill and incineration. However, only a very small fraction (about 9%) of plastic waste was recycled globally nowadays (Geyer et al., 2017). Therefore, studies developed strategies to manufacturing plastics more recyclable (Garcia and Robertson, 2017). These strategies included expanding the range of recyclable plastics, finding new low energy-consuming catalysts, and improving sorting technologies (Garcia and Robertson, 2017). Enzymatic biocatalysis, as an eco-friendly recycling alternative to conventional mechanical and chemical approaches, attracted widespread attention (Lu et al., 2022; Zhu et al., 2022). Recent studies reported that the PETase mutant called FAST-PETase could almost completely degrade 51 different PET products in 1 week, and PET was resynthesized with the recovered monomers, which indicated that a closed-loop PET recycling process was achieved and provided a feasible pathway for enzymatic recycling of PET plastics (Lu et al., 2022). However, the development of enzymatic methods in plastics recycling also faced challenges. On one hand, enzymes with excellent catalytic properties were insufficient. Although enzymes that hydrolyze ester bonds were reported, the enzyme resources were relatively scarce. Meanwhile, enzymes hydrolyzing other kinds of petro-plastics were rather scarce, such as PE and PS through linkage of monomer by carbon–carbon bonds (Lu et al., 2022). Studies demonstrated that gut microbes of some insect larvals could consume PE and PS by cleaving the carbon–carbon bonds under laboratory conditions (Stubbins et al., 2021), but the underlying mechanism of consumption and how these plastics were consumed in the natural environment were still yet not well studied. On the other hand, wild-type enzymes usually had poor thermostability and low catalytic activity and were not suitable for industrial applications. Therefore, the mining of novel enzymes and the construction or design of highly active mutants were crucial for plastic disposal and recycling.

Omics-based discovery of enzymes was a relatively new and advanced approach. Studies reported that an enzyme hydrolyzing PET was discovered from metagenome databases through *in silico* sequence-based screening (Danso et al., 2018). Additionally, esterase MGS0156 and GEN0105, with hydrolytic activity toward PLA, PCL and poly(butylene succinate-co-adipate) were obtained from environmental metagenomes based on functional metagenomics (Hajighasemi et al., 2018). Proteomics-based approach was another advanced technique for mining enzymes. Studies showed that the esterase ALC24_4107, with significant hydrolytic activity against various natural and synthetic polyester, was uncovered from *Alcanivorax* sp. 24 *via* comparative proteomic approach (Zadjelovic et al., 2020). However, currently studies about proteomics-based discovery of enzymes still mainly focused on pure microbial cultures due to the extremely challenging to obtain enzymes from complex environmental samples *via* metaproteomics (Zhu et al., 2022). Furthermore, the acquisition of high-quality protein and availability of databases for downstream bioinformatics analysis were other barriers against the development of proteomics in mining enzymes. Stable-isotope probing integrated with targeted metagenomics were contribute to locate functional microorganisms and enzymes in plastic synthesis and degradation, and improved the efficiency of enzyme mining (Thomas et al., 2018; Sander et al., 2019). In addition, various high-throughput or ultra-high-throughput screening techniques were developed to identify enzymes more efficiently and accurately, such as cell-as-compartment, micro- and pico-droplet based, and microchamber-based screening methods (Longwell et al., 2017; Bunzel et al., 2018). A recent technique called fluorescence-activated droplet sorting pipeline, which exhibited excellent throughput, sensitivity, and specificity, provided a technical support for rapid access to novel microbial and enzymes (Qiao et al., 2022).

Natural enzymes with low activity and poor thermostability were unable to meet the requirements of industrial applications, thus protein engineering was an emerging solution. Enhancing the thermostability of enzymes was one of the strategies to modify enzymes by protein engineering. The thermal stability of enzymes was essential in industrial applications, particularly for plastics with high glass transition temperature such as PET (65°C - 70°C). When the reaction temperature was close to or above the glass transition temperature, the polymeric chains in plastics would be more flexible and mobile, promoting their binding to enzymes and thus increasing degradation efficiency (Wei and Zimmermann, 2017). Recently, (Zhu et al., 2022) summarized various methods that could improve the thermal stability of plastic degradation enzymes, including the introduction of disulfide bonds or salt bridges, the formation of hydrogen bonds in the region responsible for stabilizing the enzyme structure, the introduction of proline residues, and glycan moiety. After the modifications, the Tm value of the enzymes increased by 3.7°C to 10°C. The mutation of *T. fusca* cutinase (Q132A/T101A) with larger space and higher hydrophobicity showed a significant increased efficiency for PET hydrolysis (Silva et al., 2011). Additionally, various strategies were also proposed to improve catalytic efficiency by promoting enzyme-substrate interaction, including modification of enzyme surface properties (e.g., electrostatic and/or hydrophobic properties) (Hiraishi et al., 2010; Miyakawa et al., 2015), and fusion of binding accessory to the enzyme surface (Armenta et al., 2017). However, the current methods to design mutation sites were usually empirical and uncertain, and the enzyme structure–function relationship was still not well investigated, which made challenges to accurately predict the potential beneficial mutations (Zhu et al., 2022). However, protein engineering will be greatly improved with the rapid development of computer-assisted modeling and simulation techniques. Recently, a structure-based, machine learning algorithm was successfully used to obtain the FAST-PETase, which was a mutation of PETase and exhibited superior PET-hydrolytic activity than wild-type and engineered alternatives at 30–50°C (Lu et al., 2022). Similarly, machine learning methods such as AlphaFold2 (Varadi et al., 2022), ProBound (Rube et al., 2022), RoseTTAFold (Sala et al., 2022), showed great potential in deciphering structure–function relationship and in precisely guiding protein engineering.

## Conclusion

Plastics are one of the great inventions of humans, while also bring great environment and health challenges. Bio-plastics are the mainstream of the plastic industry in the future by realizing the sustainable application of plastic products and avoiding the environmental hazards of petro-plastics. This review systematically summarized the materials for bio-plastics production, the sequences, structures and catalytic mechanisms of the related key functional enzymes for bio-plastics polymerization, and the studies about enzymatic degradation of these bio-polymers together with the petro-plastic PET and the plasticizer PAEs, the respective representative of petro-plastics and plasticizers. It is one of the key points to lead the development of plastic industry by establishing methods for efficient enzymatic ester bond synthesis or degradation in the future. This review provides a reference for the synthesis or degradation of plastic materials, and sustainable usage of bio-plastics from a new perspective of ester bond catalyzing enzymology.

## Author contributions

JL: data curation and writing – original draft. HH: data curation. ML: data curation. YX: data curation, writing – original draft, and funding acquisition. XL: writing – review and editing. BS: Writing – review and editing. All authors contributed to the article and approved the submitted version.

## Funding

This work was supported by National Natural Science Foundation of China (Nos. 32072165 and 31801467).

## Conflict of interest

The authors declare that the research was conducted in the absence of any commercial or financial relationships that could be construed as a potential conflict of interest.

## Publisher’s note

All claims expressed in this article are solely those of the authors and do not necessarily represent those of their affiliated organizations, or those of the publisher, the editors and the reviewers. Any product that may be evaluated in this article, or claim that may be made by its manufacturer, is not guaranteed or endorsed by the publisher.

## Footnotes

^1^Data available from https://www.spglobal.com/commodityinsights/en/ci/products/plasticizers-chemical-economics-handbook.html
