# Supplementary material for: Enzyme catalyzes ester bond synthesis and hydrolysis: The key step for sustainable usage of plastics
Source: Front Microbiol. 2023 Jan 12;13:1113705. doi: 10.3389/fmicb.2022.1113705 (PMC9878459; doi:10.3389/fmicb.2022.1113705)
Supplement: Supplementary file 1 [file Data_Sheet_1.docx]

Supplementary information for

**Enzyme catalyzes ester bond synthesis and hydrolysis: the key step for sustainable usage of plastics**

Jinghui Lai^1^, Huiqin Huang^1^, Mengwei Lin^1^, Youqiang Xu^1,^*, Xiuting Li^1,2^, Baoguo Sun^1,2^

^1^ Key Laboratory of Brewing Microbiology and Enzymatic Molecular Engineering of China General Chamber of Commence, Beijing Technology and Business University, Beijing 100048, China.

^2^ Key Laboratory of Brewing Molecular Engineering of China Light Industry, Beijing Technology and Business University, Beijing 100048, China.

*** Correspondence:**

Y. Xu, E-mail: [xuyouqiang@btbu.edu.cn](mailto:xuyouqiang@btbu.edu.cn)

Beijing Technology & Business University. No. 33, Fucheng Road, Haidian District, Beijing 100048, China


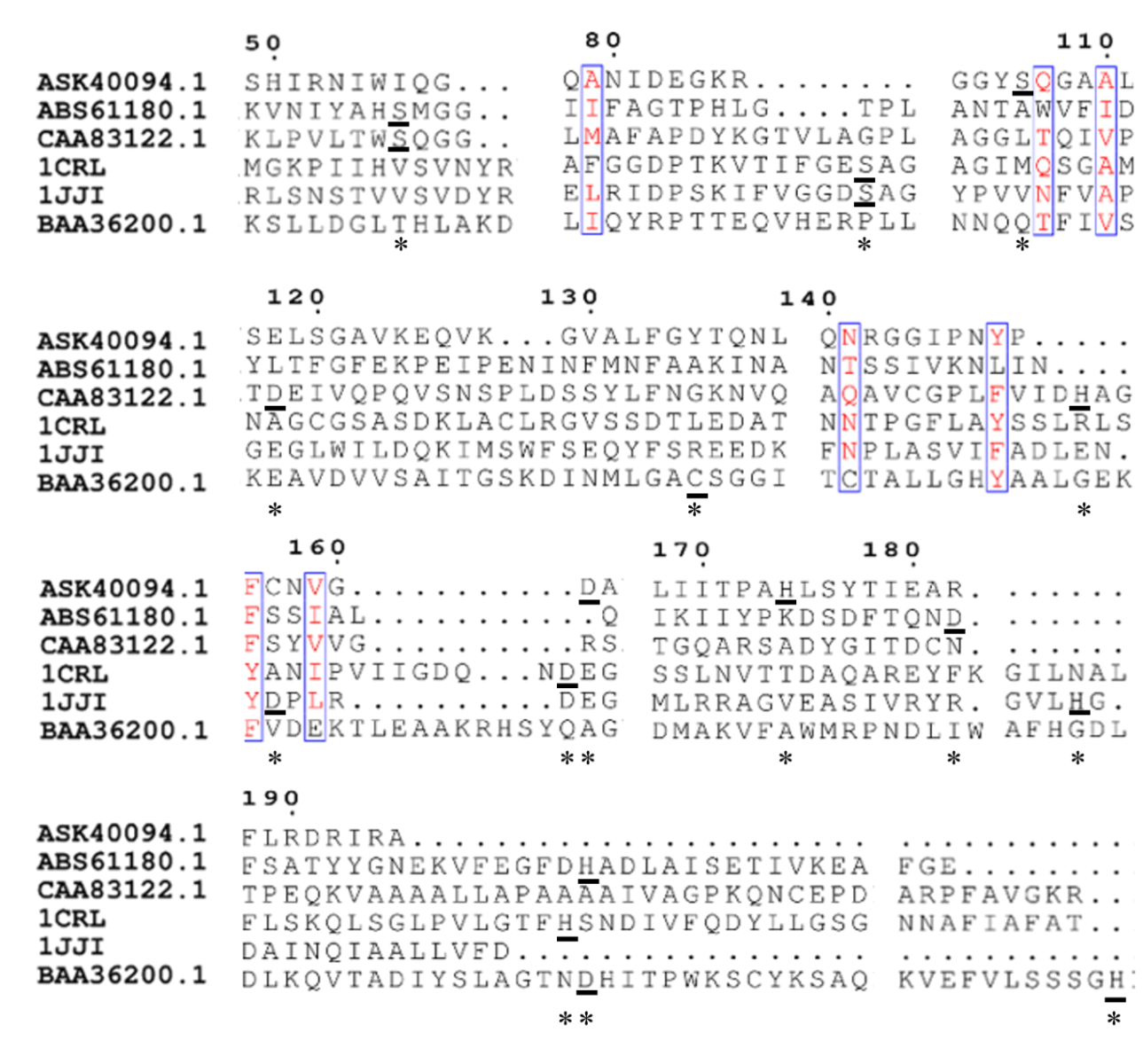


Figure S1. Sequence alignment of enzymes catalyzing the synthesis of PLA, PCL, PLGA, PES, PEF and PBS.

“*” the amino acid residue forming the catalytic triad.


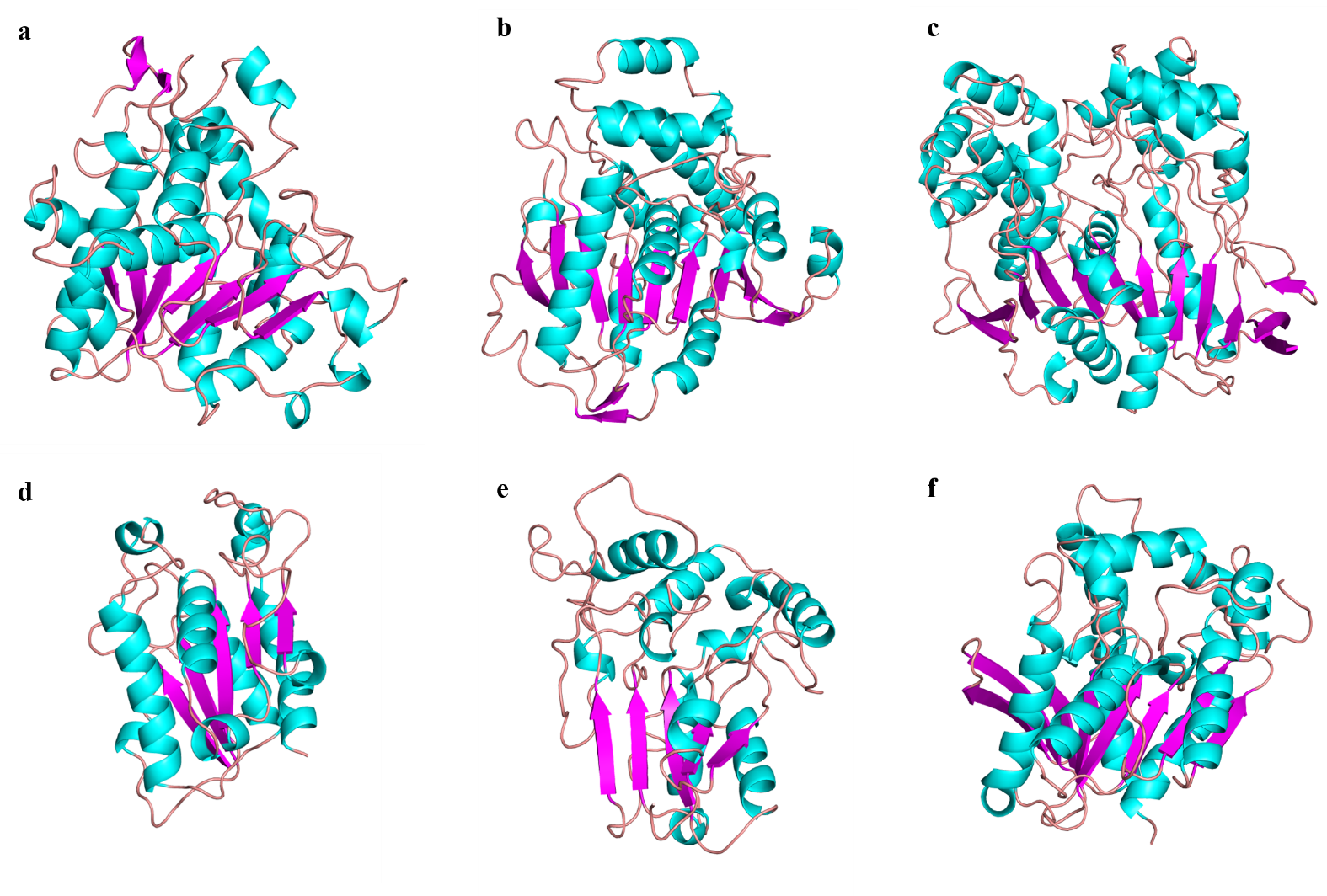


Figure S2. Homologous modeling of synthase.

(a) lipase from *Candida antarctica* (GenBank number CAA83122.1), (b) LA polymerization enzyme from *Pseudomonas* sp. 61-3 (GenBank number BAA36200.1, S325T/Q481K), (c) lipase from *Candida rugosa* (PDB ID 1CRL), (d) cutinase from *Humicola insolens* (GenBank number ASK40094.1), (e) lipase from *Fervidobacterium nodosum* Rt17-B1 (GenBank number: ABS61180.1), (f) carboxylesterase from *Archaeoglobus fulgidus* (PDB ID 1JJI).


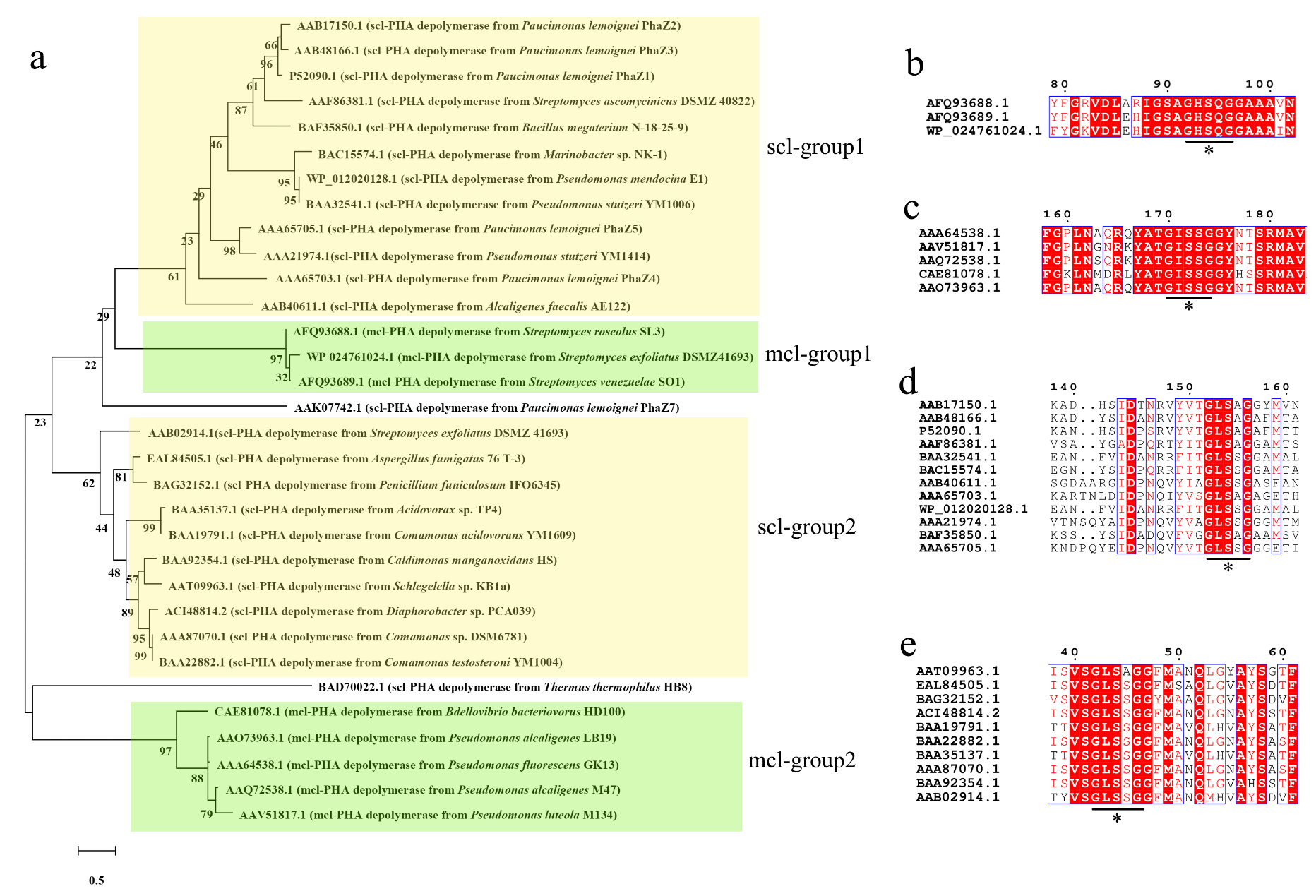


Figure S3. Sequence analysis of PHA depolymerase.

(a) The phylogenetic tree of PHA depolymerase. (b) Sequence alignment of Group 1 mcl-PHA depolymerases, (c) Sequence alignment of Group 2 mcl-PHA depolymerases. (d) Sequence alignment of Group 1 scl-PHA depolymerases. (e) Sequence alignment of Group 2 scl-PHA depolymerases. “*” Conservative amino acid.


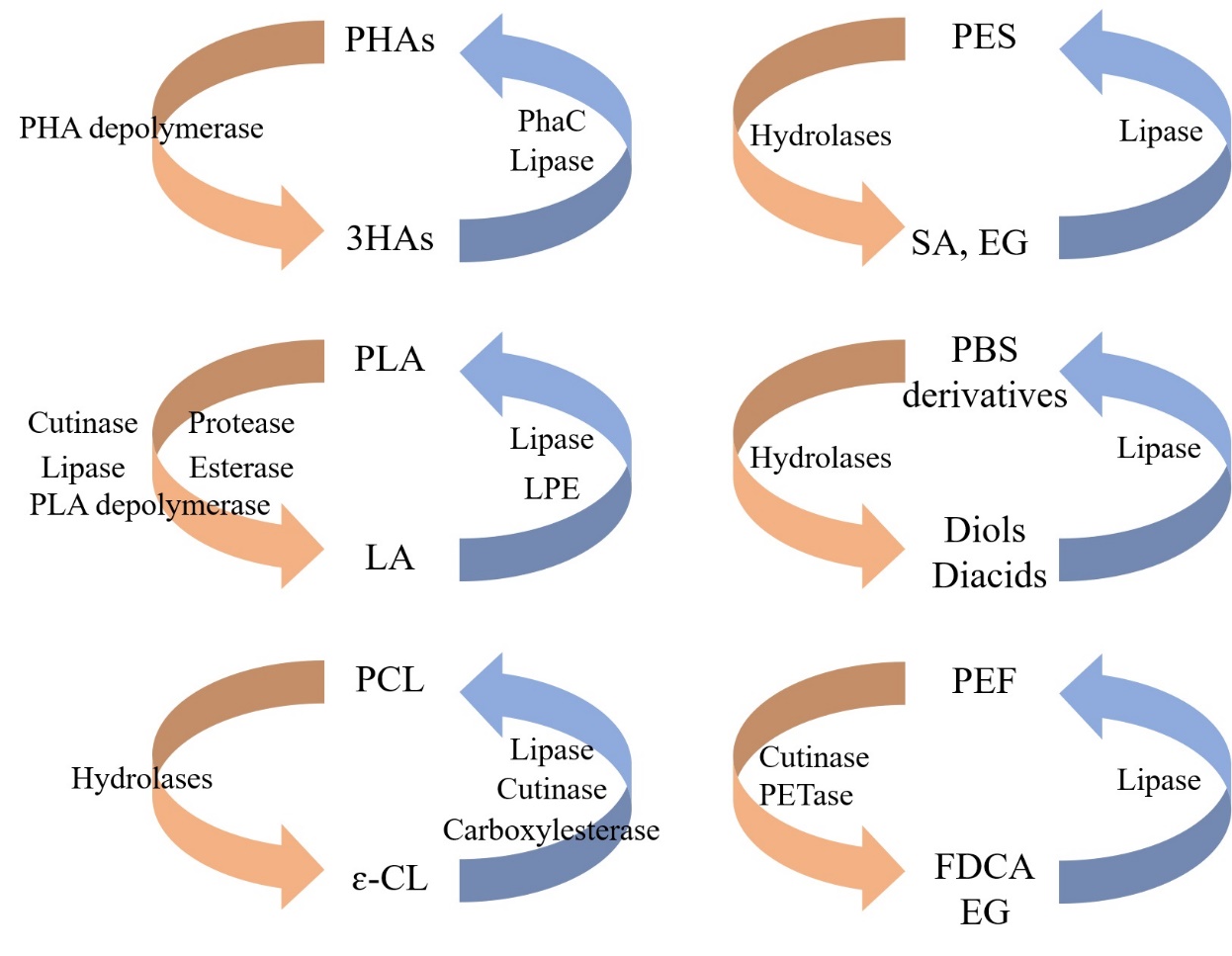


Figure S4. The summary of enzymatic synthesis and degradation of bio-plastics.

PHAs, polyhydroxyalkanoates; 3Has, 3-hydroxyalkanoates; PLA, polylactic acid; LA, lactic acid; PCL, polycaprolactone; ɛ-CL, ɛ-caprolactone; PES, poly(ethylene succinate); SA, succinic acid; EG, ethylene glycol; PBS, poly (butylene succinate); PEF, poly(ethylene furanoate); FDCA, furandicarboxylic acid; PhaC, PHA synthase; LPE, LA polymerization enzyme.
